# Distinct ZIKV strain signatures and type I IFN modulation reveal a protective role of brain endothelial interferon signaling *in vitro* and *in vivo*

**DOI:** 10.3389/fcimb.2025.1726007

**Published:** 2025-12-03

**Authors:** Luan Rocha Lima, Yasmin Mucunã Mustafá, Paula Luize Camargos Fonseca, Sharton Vinícius Antunes Coelho, Pierina Lorencini Parisi, Camila Lopes Simeoni, Lana Monteiro Meuren, Bruno Braz Bezerra, Nathane Cunha Mebus-Antunes, Flavio Matassoli, Jose Luiz Proença-Modena, Renato Santana Aguiar, Luciana Barros de Arruda

**Affiliations:** 1 Departamento de Virologia, Instituto de Microbiologia Paulo de Góes, Universidade Federal do Rio de Janeiro (UFRJ), Rio de Janeiro, RJ, Brazil; 2 Departamento de Genética, Ecologia e Evolução, Instituto de Ciências Biológicas, Universidade Federal de Minas Gerais, Belo Horizonte, Brazil; 3 Departamento de Genética, Microbiologia e Imunologia, Instituto de Biologia, Universidade Estadual de Campinas (UNICAMP), Campinas, SP, Brazil; 4 Instituto de Bioquímica Médica Leopoldo De Meis (IBqM), Universidade Federal do Rio de Janeiro (UFRJ), Rio de Janeiro, RJ, Brazil; 5 Laboratory of Immunoregulation, National Institute of Allergy and Infectious Diseases (NIAID), National Institutes of Health, Bethesda, MD, United States; 6 Instituto D’OR de Pesquisa e Ensino, Rio de Janeiro, Rio de Janeiro, Brazil

**Keywords:** Zika virus, interferon, endothelial cells, blood brain barrier, neuroinvasion

## Abstract

**Introduction:**

Zika virus (ZIKV) infection has been associated with neurological syndromes, particularly during outbreaks caused by Asian lineage strains. However, experimental models suggest that African strains may exhibit an equal or more virulent profile. Neuroinvasion by systemic viruses often requires crossing the blood–brain barrier (BBB), which disruption amplifies viral dissemination and neuropathology. Type I interferons (IFNs) are key to restricting ZIKV replication, but their specific role in preserving BBB integrity remains poorly defined.

**Methods:**

Here, we used human brain microvascular endothelial cells (HBMECs) as a simplified BBB model to compare transcriptional responses and IFN modulation following infection with either the African prototype strain ZIKV_MR766_ or the Asian epidemic strain ZIKV_PE243_. The role of endothelial cell–mediated IFN responses was further assessed *in vivo* by intravascular inoculation of mice with endothelial-specific IFNAR depletion using ZIKV_MR766_.

**Results and discussion:**

Infection of HBMEC with ZIKV_MR766_ triggered a greater number and broader range of differentially expressed genes, especially ones associated with interferon signaling and translational pathways, whereas ZIKV_PE243_-infected samples clustered closer to non-infected ones. ZIKV_MR766_ infection also resulted in higher viral titers and faster dissemination across endothelial monolayers. Both strains induced IFN-β expression but suppressed downstream IFN signaling by reducing STAT1 phosphorylation and promoting STAT2 degradation, with these effects being more pronounced for ZIKV_MR766_. Despite these evasion mechanisms, neutralization assays revealed that endothelial cells-derived IFNs production and response partially restricted viral replication, preserved HBMEC viability, and protected against barrier disruption, with ZIKV_PE243_ showing greater sensitivity to IFN-β. Importantly, *in vivo* infection of mice lacking endothelial IFNAR signaling resulted in elevated CNS viral load and increased lethality following ZIKV_MR766_ infection, underscoring the pivotal role of endothelial IFN responses in viral control, maintenance of BBB integrity, and protection against neuroinvasion.

## 1 Introduction

Zika virus (ZIKV), recently renamed *Orthoflavivirus zikaense* by ICTV, is a single-stranded RNA virus of the *Flaviviridae* family. First isolated in Uganda in 1947 (Dick et al., 1952; Simpson, 1964), it circulated silently in Africa and Southeast Asia for decades (Saba Villarroel et al., 2024; WHO, 2016), until outbreaks in the Pacific, in 2007 (Duffy et al., 2009; Cao-Lormeau et al., 2014; rev in Weaver et al., 2016), and the Americas, in 2015. Different forms of transmission and association with neuropathologies were evidenced during this period (Schuler-Faccini et al., 2016; de Oliveira et al., 2017; Musso et al., 2019). Two distinct lineages, African and Asian, have been described, with the latter linked to recent epidemics (Haddow et al., 2012; Liu et al., 2019). Although incidence has declined, ZIKV remains endemic in tropical regions (Saba Villarroel et al., 2024; Lackritz et al., 2025), underscoring the importance of continuous surveillance and comparative studies between viral lineages to better understand pathogenesis.

ZIKV infection is usually associated to a mild febrile disease, however, a percentage of infected individuals develop neurologic manifestations, particularly, after vertical transmission (Brasil et al., 2016; Melo et al., 2016; Miranda-Filho et al., 2016). Congenital Zika syndrome (CZS) encompasses a broader spectrum of abnormalities, including cerebral atrophy, intracranial calcifications, ocular anomalies, and arthrogryposis (Brasil et al., 2016; Melo et al., 2016). In experimental models, impaired brain development by ZIKV was associated with abnormal differentiation and death of neural progenitor cells, massive neuronal death, and altered vascular density and diameter, resulting in permeable blood brain barrier (BBB) (Garcez et al., 2016; Li et al., 2016; Shao et al., 2016). Although less frequent, ZIKV can also cause meningitis and meningoencephalitis in adults, which are characterized by cortical and subcortical lesions, temporal lobes hyperintensities, leukocytosis and increased protein levels in the cerebrospinal fluid (CSF) (Carteaux et al., 2016; da Silva et al., 2017). Importantly, the neurotropic and neuroinvasive nature of ZIKV is evidenced by detection of viral RNA or particles in the brains and CSF of fetuses, stillborns, and adults (Mlakar et al., 2016; Carteaux et al., 2016).

Neuroinvasion by systemic viruses often involves virus crossing through the BBB, a complex structure composed of microvascular endothelial cells associated with astrocytes, microglia, and pericytes, which regulates the traffic of cells, solutes, and pathogens into the central nervous system (CNS) (rev in Abbott et al., 2010; Kadry et al., 2020). BBB dysfunction has been implicated as a major determinant of neurological outcomes of multiple viral infections, including herpesviruses, HIV, SARS-CoV-2, and several arboviruses (rev in Mustafa et al., 2019; Boardman et al., 2025). Nonetheless, the molecular pathways through which virus replication and endothelial cell activation impacts BBB integrity during ZIKV remain poorly characterized. So far, only Asian genotype isolates have been linked to congenital and neurological disorders (Musso et al., 2019; Liu et al., 2019). However, experimental models have demonstrated that both lineages can be neuroinvasive, with isolates from African lineages often showing greater pathogenicity (Anfasa et al., 2017; Papa et al., 2017; Bowen et al., 2017; Shao et al., 2017; Tripathi et al., 2017; Lucas et al., 2018; Smith et al., 2017). Therefore, investigating the interaction between ZIKV and the BBB is critical to understanding the mechanisms of ZIKV neuropathogenesis.

Type I interferons play a key role in restricting ZIKV replication in various experimental models (Lazear et al., 2016; Grant et al., 2016; Bayer et al., 2016). While wild-type immunocompetent adult mice are resistant to ZIKV, disruption of type I IFN signaling, through deletion of type I IFN receptor (IFNAR), signal transducer and activator of transcription (STAT1), or interferon regulatory factors (IRF), renders them highly susceptible to viral replication and disease (Dowall et al., 2016; Lazear et al., 2016; Kamiyama et al., 2017). In contrast, ZIKV efficiently replicates in human fibroblasts, monocytes, dendritic cells, and endothelial cells, despite IFN production following PRR activation (Hamel et al., 2015; Papa et al., 2017; Chazal et al., 2018; Hertzog et al., 2018; Luo et al., 2018). Immune evasion strategies such as STAT2 degradation by ZIKV-NS5, reduction of STAT1/2 phosphorylation, and inhibition of IFN by other viral proteins were reported (Grant et al., 2016; Wu et al., 2017; Hertzog et al., 2018; Xia et al., 2018; Lin et al., 2019; Li et al., 2020; Shu et al., 2021; Airo et al., 2022). Also, translational control by lineage-specific upstream open reading frames (uORFs) in the 5′UTR (Lefevre et al., 2024) may further sustain viral protein synthesis under IFN-induced stress. However, it is unclear whether differential modulation of IFN signaling contributes to strain-specific neuroinvasiveness. Indeed, the role of type I IFN signaling in brain endothelial cells, particularly at the BBB, remains poorly understood, not only during ZIKV infection, but also across other virus-induced neuropathological disease.

Human brain microvascular endothelial cell lines (HBMECs) are a simplified model of the BBB that is widely employed in studies of viral infection (Rust et al., 2012; da Conceição et al., 2013; Motta et al., 2023). We and others have demonstrated that HBMECs are permissive to ZIKV infection (Papa et al., 2017; Mladinich et al., 2017), which leads to the induction of type I and III IFNs and proinflammatory mediators (Papa et al., 2017; Mladinich et al., 2017). Notably, Asian lineage isolates (e.g., PE243, PRVABC59) did not induce marked cytopathic effect (CPE) or permeability changes, whereas the African prototype MR766 caused pronounced CPE. Additionally, systemic infection of adult IFNAR-deficient mice with ZIKV_MR766_, but not ZIKV_PE243_, resulted in a lethal outcome, with disrupted BBB (Lucas et al., 2018).

Considering the central role of endothelial cells as the structural and functional core of the BBB, and the limited understanding of how IFN responses and viral replication affect these cells relative to other systems, here, we compared the infection dynamics of ZIKV_MR766_ and ZIKV_PE243_ in HBMECs, aiming to identify molecular differences that could explain the distinct virulence profiles previously observed. To achieve this, we combined transcriptomic and functional approaches, ranging from global expression profiling in HBMECs to targeted *in vitro* and *in vivo* validation assays. Transcriptomic alterations induced by ZIKV_MR766_ infection in HBMECs were markedly more extensive than those triggered by ZIKV_PE243_, particularly in pathways related to interferon-mediated antiviral responses and host translational regulation. Functional validation demonstrated that, although ZIKV_MR766_ induces higher IFN-β levels, it remains more resistant to the antiviral response. Nevertheless, endogenous type I IFN partially limited viral replication and preserved HBMEC viability and barrier integrity. *In vivo*, mice with endothelial-restricted IFNAR-depletion succumbed to ZIKV_MR766_ infection, highlighting the critical role of type I IFN signaling in endothelial cells for controlling viral replication and preserving BBB integrity.

## 2 Materials and methods

### 2.1 Cells

Culture medium and supplements were acquired from Thermo Fisher Scientific Inc. (Pittsburgh, PA, USA). Vero cells (ATCC-CCL81) were cultured in Dulbecco’s modified Eagle’s medium (DMEM) supplemented with 5% fetal bovine serum (FBS). BHK-21 cells (ATCC^®^ CCL-10) were cultured in alpha minimum essential medium (α-MEM) supplemented with 10% FBS. *Aedes albopictus* clone C6/36 cells (ATCC-CLR1660) were cultured in Leibovitz (L15) medium supplemented with 5% FBS, 0.3% tryptose phosphate broth, 0.2% sodium bicarbonate, 0.2 mM non-essential amino acids, and 0.75% L-glutamine. Human brain microvascular endothelial cells (HBMEC) (Nikolskaia et al., 2006) were kindly provided by Dr. Dennis J. Grab (Uniformed Services University of the Health Sciences, Bethesda, MD). The cells were cultured in DMEM, supplemented with 10% FBS. HBMECpISRE_luc_ reporter cells, kindly provided by Dr. Laura Gil (IpqAM, FIOCRUZ, Recife, PE, Brazil), were generated from stable transfection of HBMEC with the pISRE-Luc-Hygro reporter vector containing a pISRE-Luc in the NdeI-Bst1107 site (Stratagene, La Jolla, CA) cloned into a pCEP4 vector (Thermo Fisher Scientific). Cells were grown in DMEM, supplemented with 20% FBS, 1% L-glutamine (GlutaMAX), and hygromycin B (50 mg/mL). All cells were maintained at 37°C with a 5% CO_2_ atmosphere, except for C6/36 cells, which were maintained at 28°C in the BOD incubator.

### 2.2 Virus

ZIKA strains MR766 (ZIKV_MR766;_ ATCC VR1838); and PE243 (ZIKV_PE243_; gene bank ref. number KX197192) were kindly provided by Dr. Amilcar Tanuri (Universidade Federal do Rio de Janeiro) and Dr. Ernesto T.A. Marques Jr. (Center for Vaccine Research, University of Pittsburgh, PA), respectively. Stock samples were propagated in C6/36 cells, and the viral titers were determined by plaque assay in Vero cells, as previously described (Coelho et al., 2017). The viruses used in all the assays were from passages three or four. Viral inactivation was performed by 1 h UV exposure and confirmed by RT-qPCR in Vero cells. DENV serotype 2 (DENV-2), Asiatic strain 16681, was propagated in C6/36 cells, and viral titers were determined by plaque assay in BHK-21 cells, as previously described (Meuren et al., 2022). The supernatants of all infected cells were harvested, filtered, and stored at -80°C. The supernatants obtained from noninfected cell lines were used as mock control.

Genomic sequence analyses were performed using sequences deposited in the NCBI with accession codes NC_012532.1 and KX197192.1 for strains MR766 and Brazil/PE243/2015, respectively. Alignments of the 5′UTR nucleotide sequence and the NS5 and capsid protein amino acid sequences were performed to analyze variations between the ZIKV strains MR766 and PE243, using BioEdit (v7.2.5) with the ClustalW algorithm (default parameters), and validated by pairwise comparisons in Jalview (v2.11.5.0). Prediction of secondary structures of the 5’UTR was performed with the RNAfold WebServer, and compared with flaviviruses previously predicted sequences (Lodeiro et al., 2009). Access codes YP_009227196.1, YP_009227205.1, and ANC90426.1, were used for protein sequence analysis. PDB access codes 5U0B and 6C44 were used in this study, and protein structures were represented using PyMOL (v. 2.5).

### 2.3 *In vitro* infection

HBMECs were mock treated or inoculated with ZIKV at different MOIs, depending on the assay, and incubated for 2 h at 4°C for virus adsorption (0 hours post-infection - h.p.i.). The cells were then washed with phosphate-buffered saline (PBS) and maintained at 37^0^C/5%CO_2_ afterwards. In one set of experiments, the cells were treated or not with IFN-β (1000 U/mL – PeproTech, Cranbury, BJ) or poly I:C (50 µg/mL; Merck, Burlington, MA) or with a combination of neutralizing anti-IFN-β and anti-IFN-α (0.2μg/mL; Thermo Fisher Scientific Inc; Cat# 16-9978-81; #MA1-35514) just after virus adsorption. In another experimental setting, the cells were pre-treated with IFN-β for 24 h, washed, and then infected with the indicated viruses. The cells were cultured for different time periods, according to the experiment to be performed. Cell lysates and supernatants were harvested, and viral RNA, cytokines, and ISGs were analyzed by RT-qPCR, whereas the titer of released infectious particles was evaluated by plaque assay (Coelho et al., 2017).

### 2.4 RNA sequencing and differential expression analysis

HBMECs were mock-treated or infected, as described, using a MOI of 10. At 24 h.p.i., cells were harvested, and total RNA was extracted at 24 hpi using the ReliaPrep™ RNA Miniprep System (Promega), following the manufacturer’s instructions. RNA concentration and purity were determined with a Qubit 4 fluorometer (Thermo Fisher Scientific) and RNA integrity was assessed on a 4150 TapeStation System (Agilent Technologies). Only samples with RNA Integrity Number (RIN) ≥ 8.7 were included.

For library preparation, 100 ng of DNase-treated RNA from three replicates of two independent experiments per group (non-infected, ZIKV_PE243_, or ZIKV_MR766_) were used. Sequencing libraries were generated with the Illumina Ribo-Zero Plus rRNA Depletion Kit, Illumina cDNA Synthesis followed by Illumina RNA Prep Ligation. Cleanup steps employed Agencourt RNAClean XP and AMPure XP beads (Beckman Coulter). Libraries were indexed with IDT for Illumina DNA/RNA UD Indexes Set A, quantified using the QIAseq Library Quant Assay Kit (Qiagen), and their average fragment size (~350 bp) was confirmed by High Sensitivity D1000 ScreenTape (Agilent). Sequencing was performed as paired-end (200 cycles) on a NextSeq 500 platform (Illumina).

The raw data obtained from the sequencing passed through an initial quality analysis step. Low-quality sequences (Phred below 30) and Illumina adapters were trimmed with the BBDuk program (Bushnell, 2014). With the trimmed libraries, we used the Bowtie2 program (Langmead and Salzberg, 2012) to map the reads to ZIKV genome (MR766: NC_012532.1; PE243: KX197192.1). The mapped reads were used as input for the SPADES program (Bushmanova et al., 2019) to assemble the viral genome in each condition and to confirm the viral infection.

After assembling the viral genome, we also mapped the reads from each library to the *Homo Sapiens* genome (GRCh38 - GCA_000001405.29) using the STAR program and quantified them using the Salmon software (Patro et al., 2017). The output generated is a counting matrix that was used as input for the R package DESeq2 (Love et al., 2014) to identify differentially expressed genes (DEGs). Genes with a log2FC ≥1 and adjusted *padj*<0.05 were used for further analysis. The volcano plot was generated using the R package EnhancedVolcano (Blighe et al., 2024). The annotation (Ensembl) of *H. sapiens* was obtained using the R package BiomaRt (Durinck et al., 2009), and the packages ClusterProfiler (Xu et al., 2024) and enrichplot (Yu, 2025) were used to generate the Gene-Set Enrichment plots. The libraries sequenced in our study are available at SRA (www.ncbi.nlm.nih.gov/sra) under BioProject accession number PRJNA1337481 and accession numbers SAMN52321194–SAMN52321202.

### 2.5 Evaluation of ZIKV RNA, IFN and interferon-stimulated genes by quantitative RT-PCR

HBMECs were infected as described, and, after different time points, cells and supernatants were harvested, total RNA was isolated using the TRIzol reagent, and cDNA syntheses were performed with 1 μg RNA and random primers using High-Capacity cDNA Archive Kit mix, following the manufacturer’s protocol (Thermo Fisher Scientific Inc.). The cDNA samples were submitted to RT-qPCR using the TaqMan Universal Master Mix kit or PowerUp SYBR Green Master Mix kit (Thermo Fisher Scientific Inc.) to quantify ZIKV RNA or intracellular expression of IFN-β, OAS1, MX1, IFIT1, ISG15, and GAPDH, respectively. All the primers and probes are described in **Table 1**, and the reactions were carried out in the AriaMX Real-time PCR System (Agilent Technologies Inc., Santa Clara, CA). The absolute quantification of the ZIKV mRNA copy number occurred from a standard curve established through serial dilutions of a synthetic RNA transcript copy of the ZIKV 2007 sequence (Lanciotti et al., 2008) corresponding to the target fragment of amplification by the set of primers and probes used.

**Table 1:** Primers and probe sequences used for RT-qPCR assay.

Gene	Primer/Probe	Sequence (5’-3’)
ZIKV E	Sense	CCGCTGCCCAACACAAG
ZIKV E	Antisense	CCACTAACGTTCTTTTGCAGACAT
GAPDH	Sense	GTGGACCTGACCTGCCGTCT
GAPDH	Antisense	GGAGGAGTGGGTGTCGCTGT
IFN-β	Sense	TAGCACTGGCTGGAATGAGA
IFN-β	Antisense	TCCTTGGCCTTCAGGTAATG
OAS1	Sense	CAACGTCAAGAGCCTCATCC
OAS1	Antisense	TGGGCTGTGTTGAAATGTGT
Mx1	Sense	ACCTACAGCTGGCTCCTGAA
Mx1	Antisense	GCACTCAAGTCGTCAGTCCA
ISG15	Sense	TGTCGGTGTCAGAGCTGAAG
ISG15	Antisense	GCCCTTGTTATTCCTCACCA
IFIT1	Sense	TCAGGTCAAGGATAGTCTGGA
IFIT1	Antisense	AGGTTGTGTATTCCCACACTGTA
ZIKV E	**Probe**	FAM/AGCCTACCTTGACAAGCAGTCAGACACTCAA/3BHQ1

Cytokines and ISGs expression quantification were performed by the comparative CT method (ΔΔCt), using GAPDH Ct for normalization (Livak and Schmittgen, 2001).

### 2.6 Immunofluorescence analysis

HBMECs were infected with ZIKV_PE243_ or ZIK_MR766_ at the indicated MOIs, in the presence or not of anti-IFN-α/IFN-β. At 24 and 48 h.p.i., the cells were fixed with 4% formaldehyde, blocked with 3% BSA for 30 min, and permeabilized with 0.1% saponin diluted in a blocking solution for 15 min. Then, the samples were incubated with 4G2 antibody (supernatant from hybridoma clone D1-4G2-4-15; Ref.: ATCC HB-112) for 2 h, followed by AlexaFluor594-conjugated anti-mouse IgG (1 μg/mL, Ref.: A32744, Thermo Fisher Scientific Inc). DAPI was used to stain cells’ nuclei. The images were obtained by fluorescence microscopy, using OLYMPUS IX81 equipment and the frequency of ZIKV-infected cells (4G2+) was measured using ImageJ software (Version 1.54k).

### 2.7 Cell viability assay

HBMECs were infected with ZIKV_PE243_ or ZIK_MR766_ at the indicated MOI, in the presence or not of anti-IFN-α/IFN-β. After different time points, from 24 h to 96 h, cell viability was assessed using Cell-Titer Aqueous One Solution (Promega, Madison, USA), according to the manufacturer’s protocols. Absorbance readings were taken using a spectrophotometer (GloMax^®^-Promega); 1% Triton X-100 solution was used as a positive control.

### 2.8 Depletion of RIG-I and TLR3 by siRNA

HBMECs were transiently transfected with small interfering RNA (siRNA) targeting TLR-3, RIG-I, or nontargeting scrambled siRNA (Santa Cruz Biotechnology, Dallas, TX), using lipofectamine 2000 (Thermo Fisher Scientific Inc.), as previously described (Conceição et al., 2013). Receptors’ depletion was confirmed by western blotting analysis. At 48 h post-transfection, the cells were mock-treated or infected with ZIKV_MR766_ or ZIKV_PE243_ at a MOI of 1; poly I:C was used as a positive control. At 24 h.p.i., the cells were harvested, and IFN-β production was measured by RT-qPCR.

### 2.9 Western blotting

The expression of RIG-I, TLR-3, and phosphorylated and nonphosphorylated STAT-1 and STAT-2 were evaluated by western blotting. RIG-I and TLR3 were analyzed after 48 hours post-siRNA transfection. To analyze the expression and phosphorylation of STAT-1 and STAT-2, HBMECs were infected with ZIKV_MR766_, ZIKV_PE243_, or DENV-2 (MOI=1) for 24 h and, then cultured with IFN-β (1000 U/ml), or poly I:C (50 mg/ml) for additional 45 minutes. For all the western blotting assays, the cells were harvested in RIPA lysis buffer (10 mM Tris-HCl (pH 7.5) with 150 mM NaCl, 1% sodium deoxycholate, 0.1% SDS, Triton X-100 1%), supplemented a cocktail of protease inhibitor (Roche Applied Science, Germany). Protein concentration was measured using the Bradford quantification method (Bio-Rad Laboratórios Brasil Ltda, SP, Brazil), and 20 μg of each sample was reduced and denatured in Laemmli sample buffer containing 2-mercapto-ethanol heated for 5 min at 95°C. The samples were subjected to a 10% polyacrylamide gel electrophoresis (SDS-PAGE), followed by transfer to a nitrocellulose membrane (Merck). The membranes were blocked with 10% TBS containing 5% bovine serum albumin (BSA; Merck) for 1 h, and incubated with the antibodies anti-p-STAT1, anti-STAT1, anti-p-STAT2, anti-STAT2, anti-RIG-I, anti-TLR-3, and anti-β-actin (Cell Signaling Technology, Danvers, MA), overnight at 4^0^C. The membranes were washed and incubated with the respective HRP-conjugated secondary antibodies (Jackson ImmunoResearch Laboratories Inc., West Grove, PA) for 1 h and developed using the ECL™ Prime Western Blotting System kit (GE Healthcare, Boston, MA). The ratio between phosphorylated and nonphosphorylated proteins, as well as the ratio of total protein expression (sum of phosphorylated and nonphosphorylated) and β-actin, were calculated using ImageJ software.

### 2.10 Evaluation of ISRE activation by luciferase assay

HBMECpISREluc reporter cells were infected with ZIKV strains in the presence or absence of IFN-β or poly I:C. At 24 and 48 h.p.i., cells were washed with PBS, lysed with Luciferase Cell Culture Lysis Reagent (CCLR; Promega, Madison, WI), vortexed, and centrifuged at 12,000 × g for 2 min at 4°C. Supernatants were collected, and luciferase activity was measured using the Luciferase Assay Reagent substrate in a GloMax^®^ luminometer (Promega), following the manufacturer’s instructions. Results are expressed as relative luminescence units (RLU) normalized to the number of viable cells.

### 2.11 Analysis of type I IFN receptor by flow cytometry

HBMECs were infected with ZIKV_MR766_ or ZIKV_PE243_, at a MOI of 1 and, at 24 h.p.i., the cells were harvested, and the expression of IFNAR was evaluated by flow cytometry. Cells were fixed in 4% formaldehyde, permeabilized in 0.1% saponin with 2% FBS, and blocked in PBS with 2% FBS. Then, cells were incubated with rabbit anti-IFNAR1 antibody (0.25 mg/ml; Abcam, Cambridge, UK) for 1h, followed by incubation with PECy5-conjugated rabbit anti-IgG antibody (ThermoFisher Scientific Inc.) for 30 min. Cells stained in the absence of primary antibodies were used as controls. The samples were acquired in a FACSCanto™ cytometer and analyzed using FlowJo software (v10.8.1; Becton Dickson Immunocytometry System).

### 2.12 Analysis of BBB permeability and virus extravasation

HBMECs were seeded onto transwell inserts (Corning Costar, ME, USA; 0.4 µm membrane) and infected with ZIKV_PE243_ or ZIKV_MR766_ (MOI=0.1). The combination of neutralizing anti-IFN-β and anti-IFN-α was added to some wells just after adsorption. After 0, 24, 48 and 72 h.p.i. the transendothelial electric resistance (TEER) across cell monolayers were measured daily using a Voltohmmeter. Culture medium from the upper (luminal) and lower (abluminal) chambers were collected, and infectious virus particles were measured by plaque assay.

### 2.13 Mouse experiments and ethical statements

Male and female IFNAR^-/-^ (B6(Cg)-Ifnar1tm1.2Ees/J), CDH5_cre_ (B6.FVB-Tg (Cdh5-cre)7Mlia/J) (Alva et al., 2006) and Ifnar^flox/flox^ (B6(Cg)-Ifnar1tm1.1Ees/J) (named IFNAR^fl/fl^) (Prigge et al., 2015) mice were purchased from The Jackson Laboratories and housed/bred at the Animal Facility of the Institute of Biology, University of Campinas. Mice with IFNAR1 deficiency only in endothelial cells (CDH5_cre_+ IFNAR^fl/fl^) were generated by mating CDH5_cre_ and IFNAR^fl/fl^. Mice CDH5_cre(negative)_ IFNAR^fl/fl^ from the same litter were used as controls in all experiments. All animals were genotyped using tail samples digested by proteinase K as previously described (Alva et al., 2006). Animals were housed in groups of five per cage with free access to food and water, under a 12 h light/dark cycle, with controlled temperature and humidity. All procedures followed the “Principles of Laboratory Animal Care” (US National Institutes of Health) and institutional policies for animal care and usage and were approved by The Ethics Committee of Animal Care and Use from UNICAMP (protocol 4858-1/2018).

### 2.14 *In vivo* infection, tissue collection and analysis of viral load

Four-week-old mice with body weight between 11-13g, CDH5_cre(negative)_ IFNAR^fl/fl^ (12 mice) and CDH5_cre_+ IFNAR^fl/fl^ mice (27 mice) were inoculated with 30μl (2x10^5^ PFU) suspension of ZIKV_MR766_, as previously described (Lucas et al., 2018). Six mice per group were mock inoculated, as negative controls. After injection, the animals were monitored daily to assess body weight, neurological signs, and survival. Mice were euthanized at the indicated time points. In addition, mice presenting one or more physical or behavioral characteristics determined as humane endpoint (weight loss above 20%, intense lethargy or no mobility, or excessive agitation) were considered as end-stage illness or moribund, and euthanized, following the policies for animal care and usage. Dead and moribund mice were included as dead in the survival curves, and the ones found at end-stage illness were used to assess viral load in the tissues. The brains and spleens were harvested, weighed, and macerated in DMEM medium supplemented with 1% gentamicin, following the ratio of 0.2 mg of tissue to 1 μL of medium. After homogenization, centrifugation was performed at 4500 g for 5 min to remove tissue residue. Viral load in the tissues was determined by plaque assay.

### 2.15 Statistical analysis

Statistical analyses were performed using GraphPad Prism v.8 software. Differences between groups were analyzed by One-Way ANOVA or Two-Way ANOVA, followed by multiple comparisons posttests indicated in the legends; comparisons between two groups were performed by Student’s t-test for unpaired samples. IFN-β IC_50_ and IC_90_ were also calculated using GraphPad Prism after data normalization and nonlinear curve progression analyses. Values for all measurements are expressed as mean or mean ± standard deviation, and every statistical method is indicated in the corresponding legend. Analyses with p-value <0.05 were considered significant. (*) p ≤ .05; (**) p ≤ .01; (***) p ≤ .001; (****) p ≤ .0001; not significant (ns).

## 3 Results

### 3.1 Global transcript abundance and differential gene expression profiles induced by ZIKV_MR766_ and ZIKV_PE243_ replication in HBMECs

Aiming to investigate whether ZIKV_MR766_ and ZIKV_PE243_ replication could differentially impact BBB integrity, we performed comparative global gene expression and functional analyses of HBMEC infected with each strain, as a simplified model of BBB. The transcriptional landscape of ZIKV infection was assessed by RNAseq of control samples and HBMECs infected with each ZIKV strain. A MOI of 10 was chosen to synchronize infection for transcriptomic analysis, as this condition resulted in nearly all HBMECs were 4G2-positive at 24 h.p.i., with no significant cell death detected for either strain (**Supplementary Figure S1A, B**). Transcript abundance quantification in tpm (transcripts per million) revealed that ZIKV_MR766_ generated a higher number of reads compared to ZIKV_PE243_ (**Supplementary Figure S1C**). Indeed, differential expression analysis identified 121 differentially expressed genes (DEGs) in ZIKV_PE243_-infected samples compared to control, with 44 up-regulated and 77 down-regulated genes. In contrast, ZIKV_MR766_-infected samples displayed a markedly stronger transcriptional response, with a total of 285 DEGs, including 110 up-regulated and 175 down-regulated genes (**Supplementary Figure S1D**).

**Figure 1:**
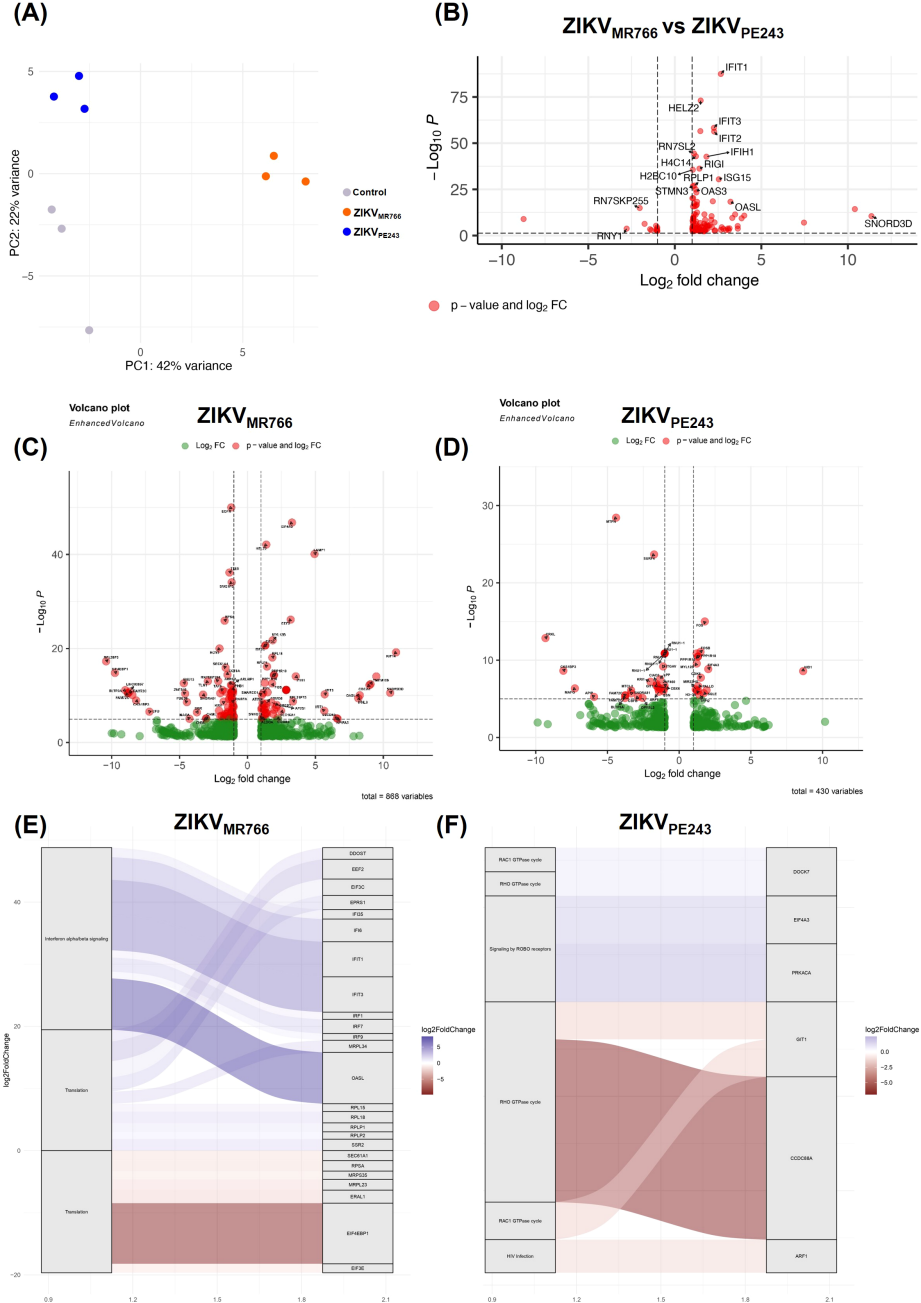
Global gene expression analysis in HBMECs infected with different ZIKV lineages. **(A)** Principal component analysis (PCA) showing control samples and cells infected with ZIKV_MR766_ and ZIKV_PE243_ strains. **(B)** Differentially expressed genes (DEGs) between ZIKV_MR766_ infection condition compared to ZIKV_PE243_ condition. **(C, D)** Volcano plots depicting DEGs in HBMECs infected with ZIKV_MR766_**(C)** and ZIKV_PE243_**(D)**, compared to control. Three biological replicates were analyzed for each condition and significantly regulated genes according to p-values and log2 fold changes are labeled in each volcano plot. **(E, F)** Differentially expressed genes associated with biological pathways during ZIKV infection compared with control condition. The plot illustrates the relation between genes and enriched pathways in infections with the ZIKV_MR766_**(E)** and ZIKV_PE243_**(F)**.

Principal component analysis (PCA) revealed three distinct clusters corresponding to the control, ZIKV_MR766_, and ZIKV_PE243_-infected samples (**Figure 1A**). Control and ZIKV_PE243_ samples clustered closely, whereas ZIKV_MR766_ showed a more divergent transcriptional profile. Volcano plots highlighted these differences, with ZIKV_MR766_ inducing substantially more DEGs (n=868) than ZIKV_PE243_ (n=430) (**Figures 1B, C**). In the ZIKV_MR766_-infected samples, numerous transcripts were significantly upregulated, including genes such as *EGR1*, *EEF1A1*, *AMP1*, and *KIF4*, which are commonly associated with cellular stress responses and viral replication. Several genes such as *PBLD*, *EBP*, and *LINC00857* were markedly downregulated, suggesting the suppression of host pathways that may hinder viral propagation. In contrast, the ZIKV_PE243_ infection resulted in a lower number of significantly dysregulated genes, though a few exhibited large fold changes, including *MD1*, *MT1P1*, and *FOSB*. Genes such as *FOS* and *RNU1–1* were significantly altered in both conditions, pointing to shared host response elements triggered by both viral lineages. To explore the overlap and specificity, we compared the sets of DEGs (**Supplementary Figure S1E**). Among upregulated genes, 94 were unique to ZIKV_MR766_, 26 to ZIKV_PE243_, and 18 shared (**Supplementary Table S1**). For downregulated genes, 162 were unique to ZIKV_MR766_, 63 to ZIKV_PE243_, and 14 were common to both (**Supplementary Table S2**).

Direct comparison of lineages (**Figure 1D**) confirmed a distinct MR766 signature, with 117 genes significantly upregulated. Among the most prominently upregulated transcripts were classical interferon-stimulated genes (ISGs), including *IFIT1, IFIT2, IFIT3, IFIH1, ISG15, OAS1*, and *OASL*, as well as *RIG-I* (DDX58), reflecting the activation of innate antiviral responses. Other notable transcripts included *HELZ2* and *RPLP1*, the latter implicated in viral translation and replication processes. *SNORD3D* also exhibited strong upregulation, suggesting potential modulation of small RNA processing during infection.

Pathway enrichment analysis (**Figures 1E, F**) also supported that MR766 predominantly activated interferon α/β signaling and translation, consistent with the upregulation of IRFs and ISGs (*IFI6, IFIT1, IFIT3, IRF1, IRF7, ISG15*) and genes involved in the translational machinery (*EIF3C, RPL15, RPL2, MRPL23*). In contrast, ZIKV_PE243_ elicited enrichment in distinct pathways, with prominent activation of RHO and RAC1 GTPase cycles, signaling by ROBO receptors, and HIV infection-related pathways. Key genes such as *CCDC88A*, *GIT1*, and *ARF1* were implicated in cytoskeletal remodeling and intracellular trafficking, which may support viral entry or replication. The genes *PRKACA* and *EIF4A3*, involved in signaling and RNA processing, respectively, were also enriched. Together, these results indicate that ZIKV_MR766_ elicits a more robust and extensive transcriptional response than ZIKV_PE243_, predominantly activating immune-related and translational processes, whereas ZIKV_PE243_ modulates pathways linked to cytoskeletal dynamics and signal transduction. These distinct patterns potentially reflect distinct viral–host interaction dynamics between the African and Asian lineages.

**Figure 2:**
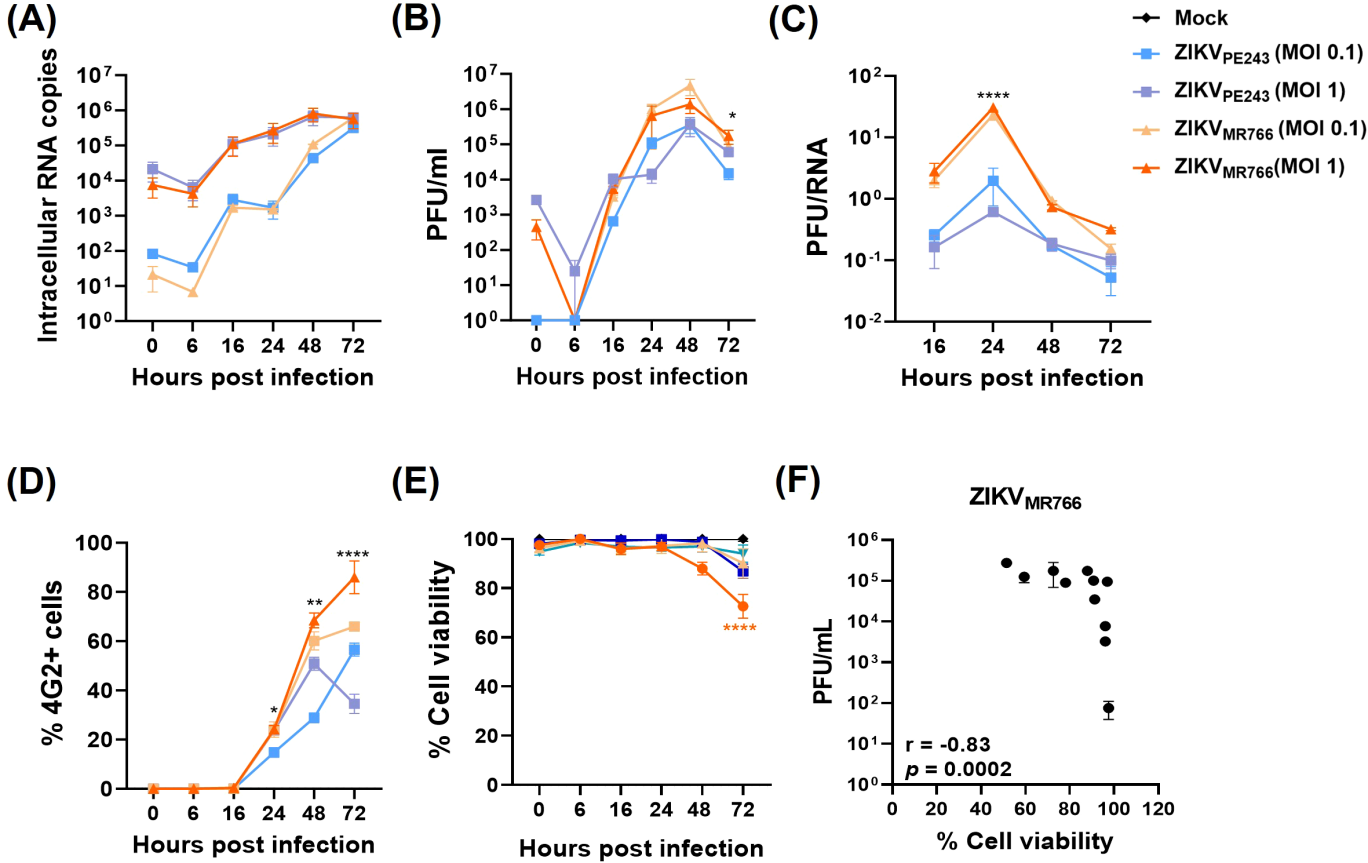
Replication, dissemination, and cytopathic effects of ZIKV strains in HBMECs. HBMECs were infected with ZIKV_PE243_ or ZIKV_MR766_, at a MOI of 1 or 0.1, and cell lysates and culture medium were collected at the indicated time points. **(A)** ZIKV mRNA copies were quantified in cell lysates by RT–qPCR. **(B)** Infectious viral titers were measured in the culture medium by plaque assay. **(C)** Ratio between PFU and viral RNA levels comparing ZIKV_PE243_ and ZIKV_MR766_. **(D)** Infected cells were stained with the 4G2 antibody, and the frequency of infected cells (%4G2^+^) was determined by immunofluorescence. **(E)** Cell viability was assessed using the CytoTox 96^®^ Non-Radioactive Cytotoxicity Assay. **(F)** Correlation between viral titers (PFU) and cell viability in HBMECs infected with ZIKV_PE243_ and ZIKV_MR766_. Data represent mean ± SD of two independent experiments. *p<0.05; **p<0.01; ***p<0.001.

To further characterize the model, we performed a series of time-course experiments using various MOIs to monitor key parameters of viral replication. No significant difference was observed in the levels of viral RNA released between the two ZIKV isolates when using the same MOI (**Figure 2A**). However, the concentration of infectious viral particles was significantly higher in cultures infected with ZIKV_MR766_ compared to those infected with ZIKV_PE243_ (**Figure 2B**). Consistently, the PFU-to-RNA ratio was significantly different between the two strains at all time points analyzed (**Figure 2C**), suggesting more efficient viral maturation by ZIKV_MR766_. Immunofluorescence analysis of 4G2-positive cells over time revealed a faster spread of ZIKV_MR766_ infection relative to ZIKV_PE243_ (**Figure 2D**), likely due to the earlier release of infectious particles. While ZIKV_PE243_-infected HBMECs remained viable throughout the infection course, ZIKV_MR766_ infection induced a modest but significant reduction in cell viability from 72 h.p.i. (**Figure 2E**), which correlated with viral replication (**Figure 2F**).

### 3.2 ZIKV_MR766_ evades IFN response more efficiently, through reduction of STAT1 phosphorylation and degradation of STAT2

**Figure 3:**
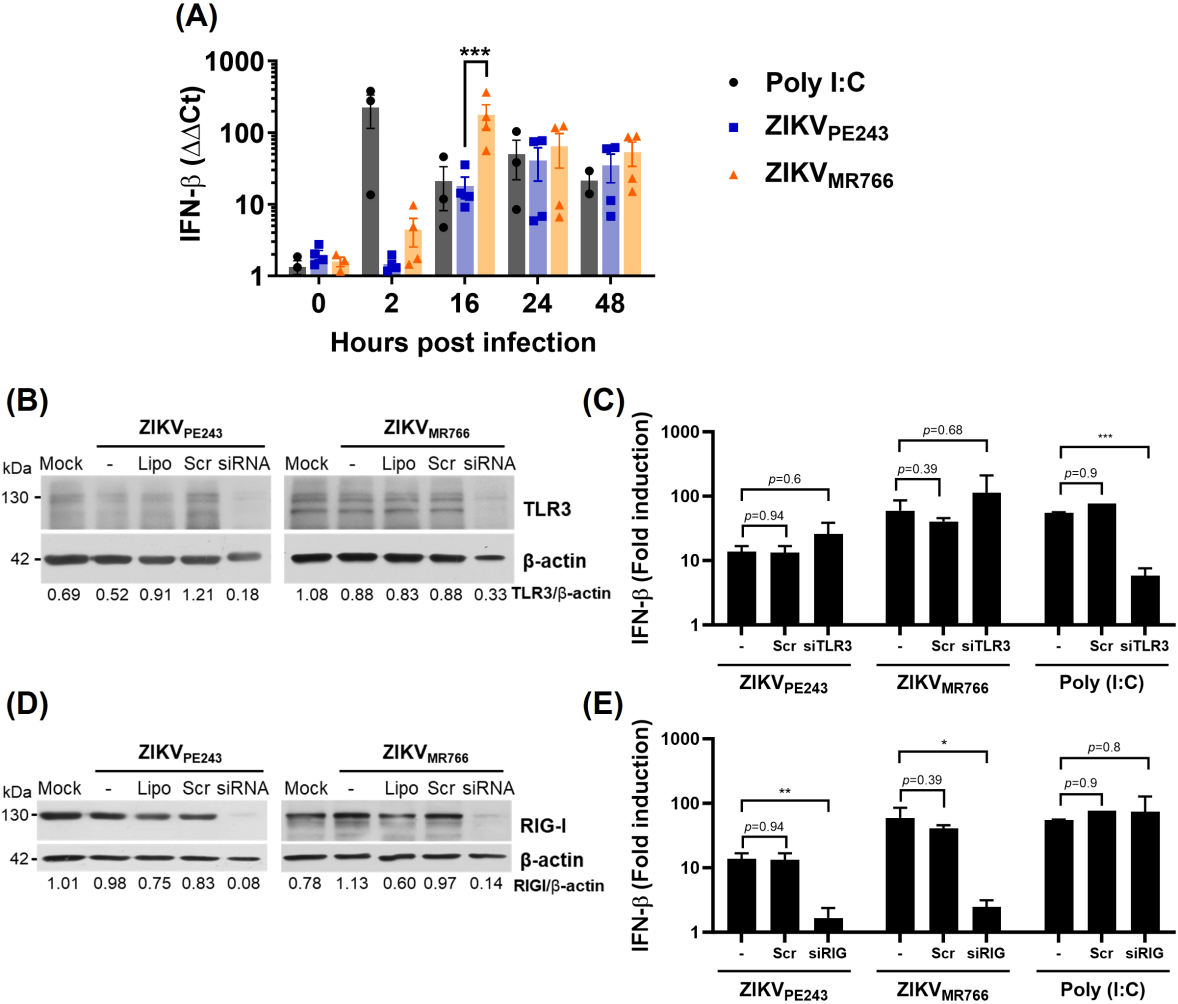
ZIKV induces RIG-I-dependent IFN-β production by HBMECs. **(A)** HBMECs were mock-treated or infected with ZIKV_PE243_ or ZIKV_MR766_ (MOI=1); poly (I:C) was used as positive control. The cells were harvested at the indicated time points and IFN-β mRNA was measured by RT-qPCR. Bars represent the mean and standard deviation of seven independent experiments; statistical analyses were performed by two-way anova and Bonferroni’s multiple comparisons test. **(B-E)** Depletion of TLR3 **(B, C)** and RIG-I **(D, E)** in HBMECs were performed by specific siRNA transfection; lipofectamine only (Lipo), and scramble siRNA (Scr) were used as controls. Receptor depletion was confirmed by western blotting using anti-TLR3 **(B)** or anti-RIG-I **(D)** specific antibodies, and anti-βactin as a loading control. After 48 h, the cells were mock-treated or infected with ZIKV_PE243_ or ZIKV_MR766_ and, at 24 hpi, IFN-β expression was evaluated by RT-qPCR **(C, E)**. Statistical analysis were performed by unpaired t test; *p<0.05, **p<0.01, ***p<0.001.

Since many of the transcriptional changes observed in infected HBMECs were associated with IFN production and signaling, we next sought to investigate this pathway in greater detail. Analysis of IFN-β expression by RT-qPCR confirmed that both ZIKV strains induced IFN-β expression in HBMECs, with ZIKV_MR766_ eliciting a stronger and earlier response (**Figure 3A**). To identify the viral sensors responsible for IFN-β induction, we focused on key pattern-recognition receptors involved in RNA virus detection. While TLR3 recognizes viral dsRNA within endosomal vesicles, RIG-I and MDA5 act as cytoplasmic sensors that frequently function in a redundant manner, as reported in ZIKV infection models (Lin et al., 2019). Therefore, we selected TLR3 and RIG-I for depletion as representatives of endosomal and cytoplasmic sensing pathways, respectively. Poly I:C was added to the cultures, without transfection, as a positive control for TLR3, but not RIG-I activation. As expected, TLR3 depletion, but not RIG-I, significantly reduced poly I:C-induced IFN-β mRNA expression. On the other hand, while TLR3 silencing did not affect ZIKV-induced IFN-β transcription (**Figures 3B, C**), depletion of RIG-I almost abolished IFN-β expression in cells infected with either ZIKV_PE243_ (88% inhibition; *p*=0.008) or ZIKV_MR766_ (94% inhibition; *p*=0.0004) (**Figures 3D, E**), indicating RIG-I as a major RNA sensor related to this pathway.

**Figure 4:**
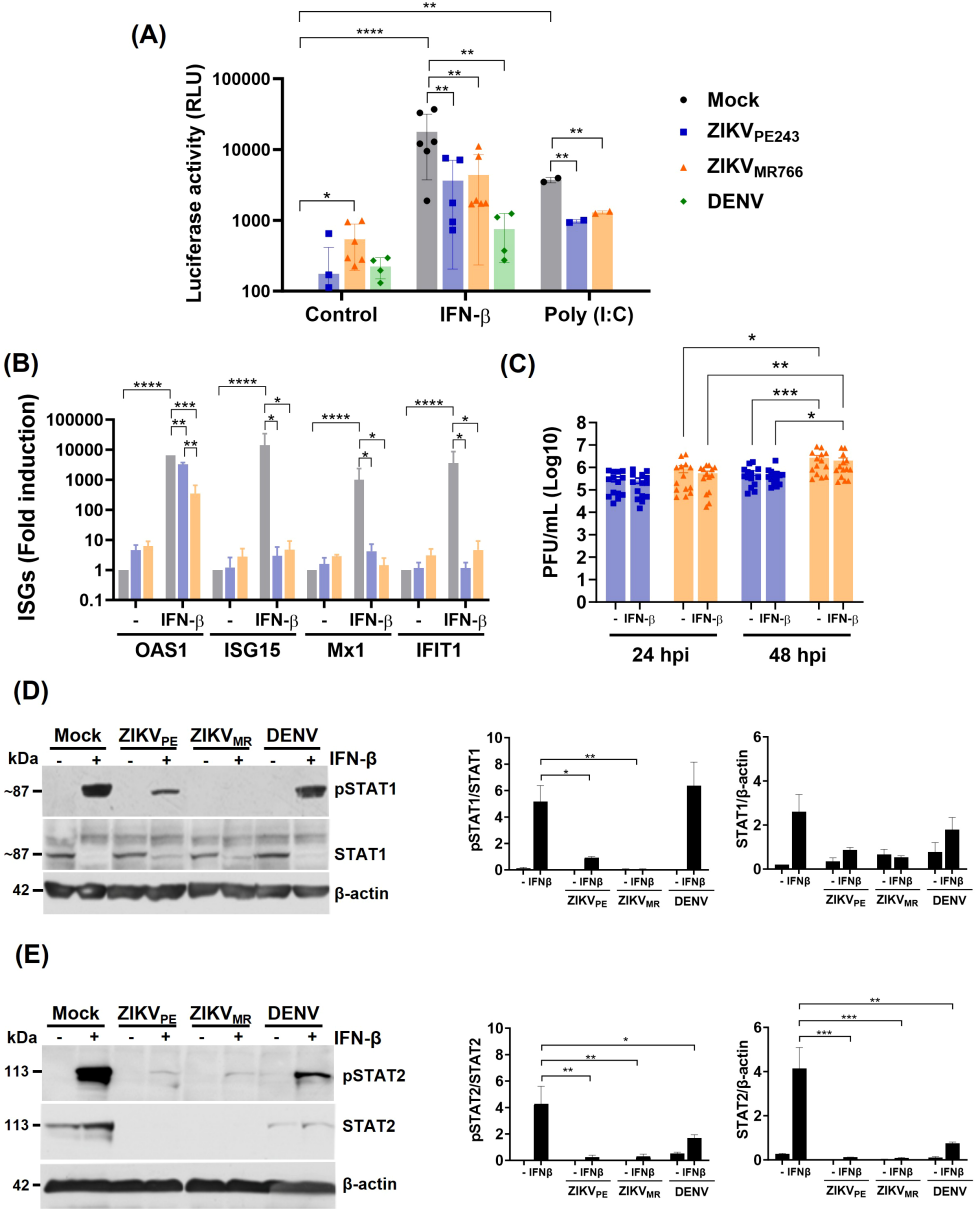
ZIKV infection inhibits IFN-β response in HBMEC. **(A)** pISRE-HBMEC-luc reporter cells were mock-treated or infected with ZIKV_PE243_, ZIKV_MR766_ or DENV-2 at a MOI of 1. Cells were treated with IFN-β or poly I:C after virus adsorption and cellular luciferase activity was measured at 24 hpi. **(B, C)** HBMECs were cultured as in **(A)**. At 24 hpi, OAS1, ISG15, MX1, and IFIT1 mRNA was assessed by RT-qPCR **(B)** and released viral titers was measured by plaque assay **(C)**. The data represent the mean and standard deviation of five independent experiments, and the statistical analyses were performed by two-way ANOVA and Dunnett’s multiple comparisons test. **(D, E)** The expression of phosphorylated or nonphosphorylated STAT1 (pSTAT1, STAT1) **(D)** and of pSTAT2 and STAT2 **(E)** were evaluated by western blotting; β-actin expression was measured as loading control. Representative membranes are demonstrated in the left panels; the ratio of pSTAT1 in relation to STAT1, as well as pSTAT2 in relation to STAT2 are indicated in the middle panels; and total STAT1 or STAT2 expression in relation to β-actin are demonstrated in right panels. Data represent mean and standard deviation of six independent experiments, and the statistical analysis were performed by one-way anova and Tukey’s multiple comparisons test; *p<0,05, **p<0,01, ***p<0,001, ****p<0,0001.

Given the efficient ZIKV replication in HBMECs, we assessed whether the viruses could evade IFN-mediated antiviral response. HBMECpISRE_luc_ reporter cells were infected with ZIKV_PE243_ or ZIKV_MR766_ at MOI 1, and, after viral adsorption, cells were treated with IFN-β or poly I:C. DENV infection was used as a control for inhibition of IFN-response (Morrison et al., 2013). Infection of HBMECs with both virus strains significantly reduced the activation of ISRE induced by either IFN-β itself or by poly I:C stimulation (**Figure 4A**). Accordingly, IFN-β-induced expression of the interferon-stimulated genes (ISG) OAS1 (*2’-5’-oligoadenylate synthetase 1*), Mx1, ISG15, and IFIT1 (*Interferon-induced protein with tetratricopeptide repeats 1*) were inhibited when the cells were previously infected with ZIKV_MR766_ or ZIKV_PE243_ (**Figure 4B**). Addition of IFN-β after infection resulted in only ~20% inhibition of viral replication, further supporting that ZIKV can efficiently counteract late IFN signaling once infection is already established (**Figure 4C**).

Viral replication did not alter IFNAR expression (**Supplementary Figure S2**) but led to reduced phosphorylation of STAT1 (**Figure 4D**), as well as both degradation and reduced phosphorylation of STAT2, similarly to DENV-infection (**Figure 4E**). Notably, inhibition of STAT activation and decreased expression of most ISGs were more pronounced in ZIKV_MR766_-infected cells (**Figures 4B, D**), prompting us to investigate potential viral determinants underlying these differences.

**Figure 5:**
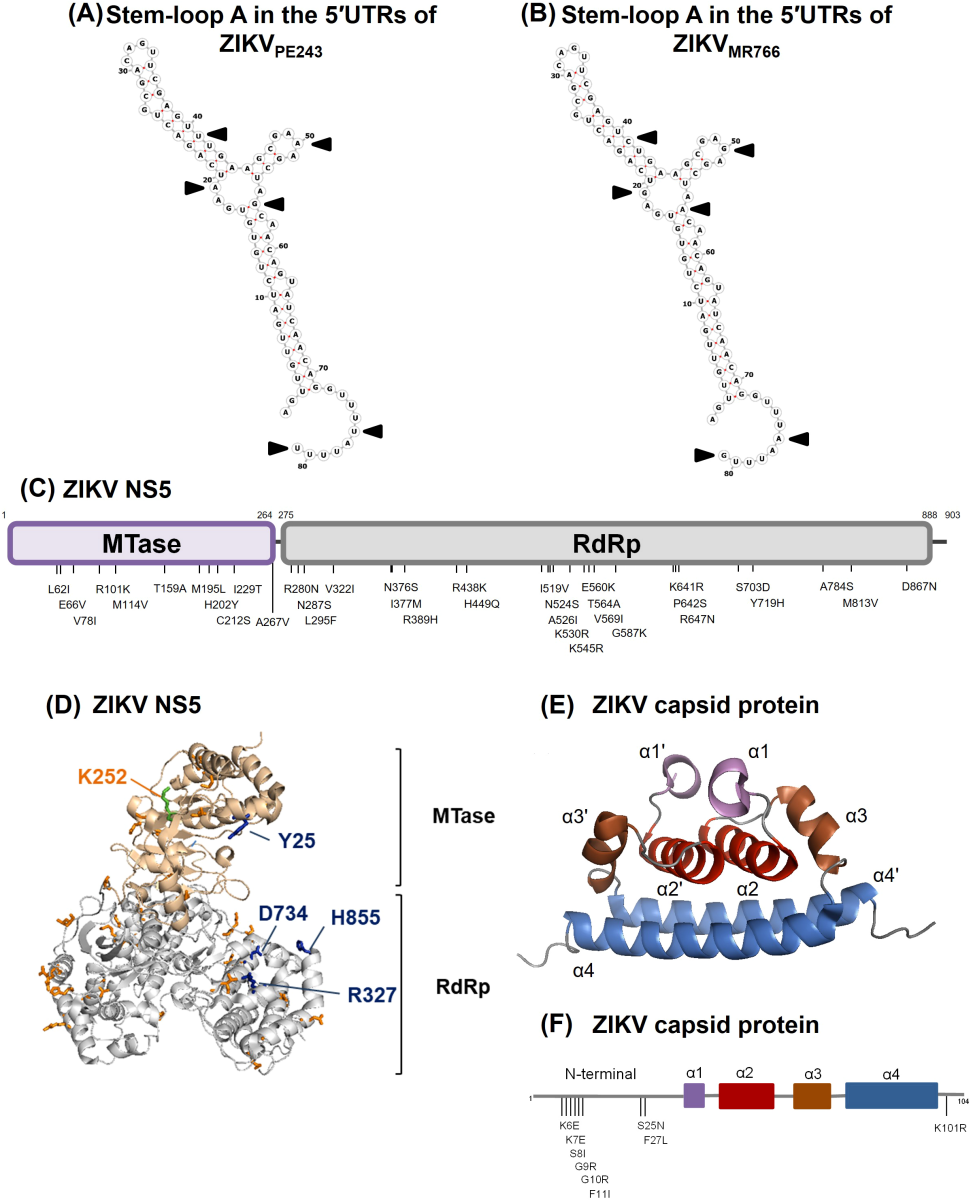
Comparison of genomic and amino acid sequences by the 5’UTR, NS5 and capsid protein from ZIKV_MR766_ (NC_012532.1) and ZIKV_PE243_ (GenBank KX197192.1). **(A, B)** Sequence and predicted secondary structure of stem-loop A in the 5′UTRs from ZIKV_PE243_**(A)** and ZIKV_MR766_**(B)**. The black arrows indicate sequence variations in the 5′-UTR. **(C)** Schematic representation of ZIKV NS5 from ZIKV_MR766_ indicating the amino acid substitutions in the NS5 from ZIKV_PE243_. MTase and RdRp domains are colored purple and gray, respectively. **(D)** Ribbon representation showing the MTase (light pink) and RdRp (gray) domains of ZIKV_MR766_ NS5. The locations of residues that differed in NS5 from ZIKV_PE243_ in the context of ZIKV_MR766_ NS5 structure (PDB, 5U0B) are shown in orange sticks. The residues Y25, R327, D734 and H855 are highlighted in blue sticks, and the K252 residue (green stick) is labeled in orange. **(E)** Ribbon representation showing the structure of the ZIKV capsid protein (PDB, 6C44), with the α-helix pairs indicated: α1/α1’ (purple), α2/α2’ (red), α3/α3’ (brown), and α4/α4’ (blue). **(F)** Schematic representation of the capsid protein from ZIKV_PE243_ indicating the amino acid substitutions from ZIKV_MR766._ The α-helices are colored according to protein structure in **(E)**.

Comparison of the ZIKV_PE243_ and ZIKV_MR766_ genomes revealed several nucleotide and amino acid differences in regions potentially linked to IFN modulation. A few nucleotide substitutions were identified in the 5′ UTR of both ZIKV strains, most of which have not been associated with IFN modulation (Chazal et al., 2018). However, we identified a specific nucleotide insertion at position 81, consistent with the lineage-specific upstream open reading frames (uORFs) previously described (Lefevre et al., 2024). These uORFs have been implicated in modulating viral translation and conferring resistance to translational arrest under stress conditions, which could enhance IFN resistance. Sequence alignment confirmed the presence of this insertion in ZIKV_PE243_ but not in ZIKV_MR766_ (**Supplementary Figure S3**). We did not find any differences in the NS5 positions Y25-R327, D734, and H855, which were previously determined to bind to STAT2 (Wang et al., 2020), as well as in the K252 SUMOylation position (Conde et al., 2020). However, a few other substitutions were detected at methyltransferase and RdRp domains, although their role in NS5-STAT2 interactions had not been investigated (**Figures 5C, D**). Amino acid substitutions were also seen when comparing the capsid sequence of ZIKV_PE243_ and ZIKV_MR766_ (**Figures 5E, F**), which could affect RIG-I ubiquitination and IFN production (Airo et al., 2022). Those include K101R substitution, which was recently associated to enhanced neurovirulence of ZIKV African strains (Song et al., 2023).

### 3.3 IFN-β partially protects HBMEC from ZIKV replication

**Figure 6:**
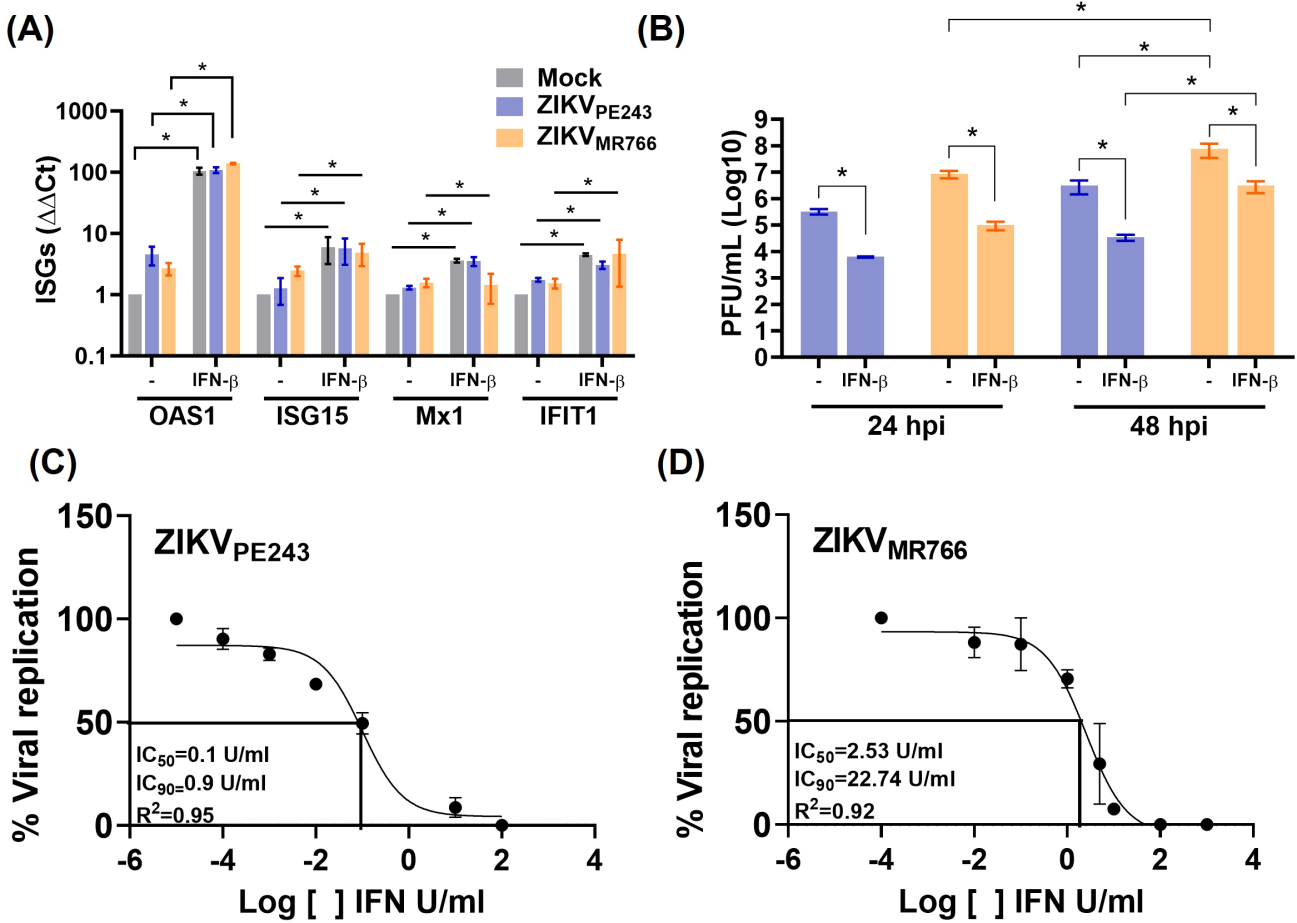
IFN-β treatment before ZIKV infection reduces viral replication. **(A, B)** HBMECs were treated with IFN-β (1000U/mL) and, after 24 h, cells were mock-treated or infected with ZIKV_PE243_ or ZIKV_MR766_ (MOI=1). Cells were harvested and mRNA expression of OAS1, ISG15, MX1, and IFIT1 were measured by RT-qPCR **(A)**. Viral titers were measured by plaque assay **(B)**. **(C, D)** HBMECs were treated with different concentration (10^-3–^10^4^ U/mL) of IFN-β for 24 hours and were then infected with ZIKV_PE243_**(C)** or ZIKV_MR766_**(D)**. At 24 hpi, the supernatants were harvested, virus titration was performed by plaque assay, and the IFN concentration inhibiting 50% and 90% of virus titer (IC_50_ and IC_90_) were calculated with GraphPad prism. The data represent mean and SD of three independent experiments. Bars indicate average and SD from three independent assays and statistical analysis were performed by two-way anova and Tukey’s multiple comparison test; *p<0.05; **p<0.01 ***p<0.001.

Despite the viral mechanisms of IFN evasion, we next assessed whether IFN-β could still confer protection to noninfected bystander endothelial cells when present prior to infection. To evaluate this, HBMECs were pretreated with the cytokine for 24 h before infection with ZIKV_PE243_ or ZIKV_MR766_. At this point, ZIKV infection did not affect IFN-β-induced ISG expression (**Figure 6A**), and viral replication was significantly reduced (**Figure 6B**). This indicates that IFN-β signaling before infection established an antiviral state in bystander cells, despite the virus’s ability to evade these responses once infection is established. To compare the sensitivity of each virus strain to IFN-β, the cells were cultured with different concentrations of the cytokine before infection, and the IC_50_ and IC_90_ were calculated. ZIKV_MR766_ showed higher resistance to IFN-β than ZIKV_PE243_, with IFN-β IC_50/90_ of 2.53/22.74 and 0.1/0.9 U/ml, respectively (**Figures 6C, D**).

**Figure 7:**
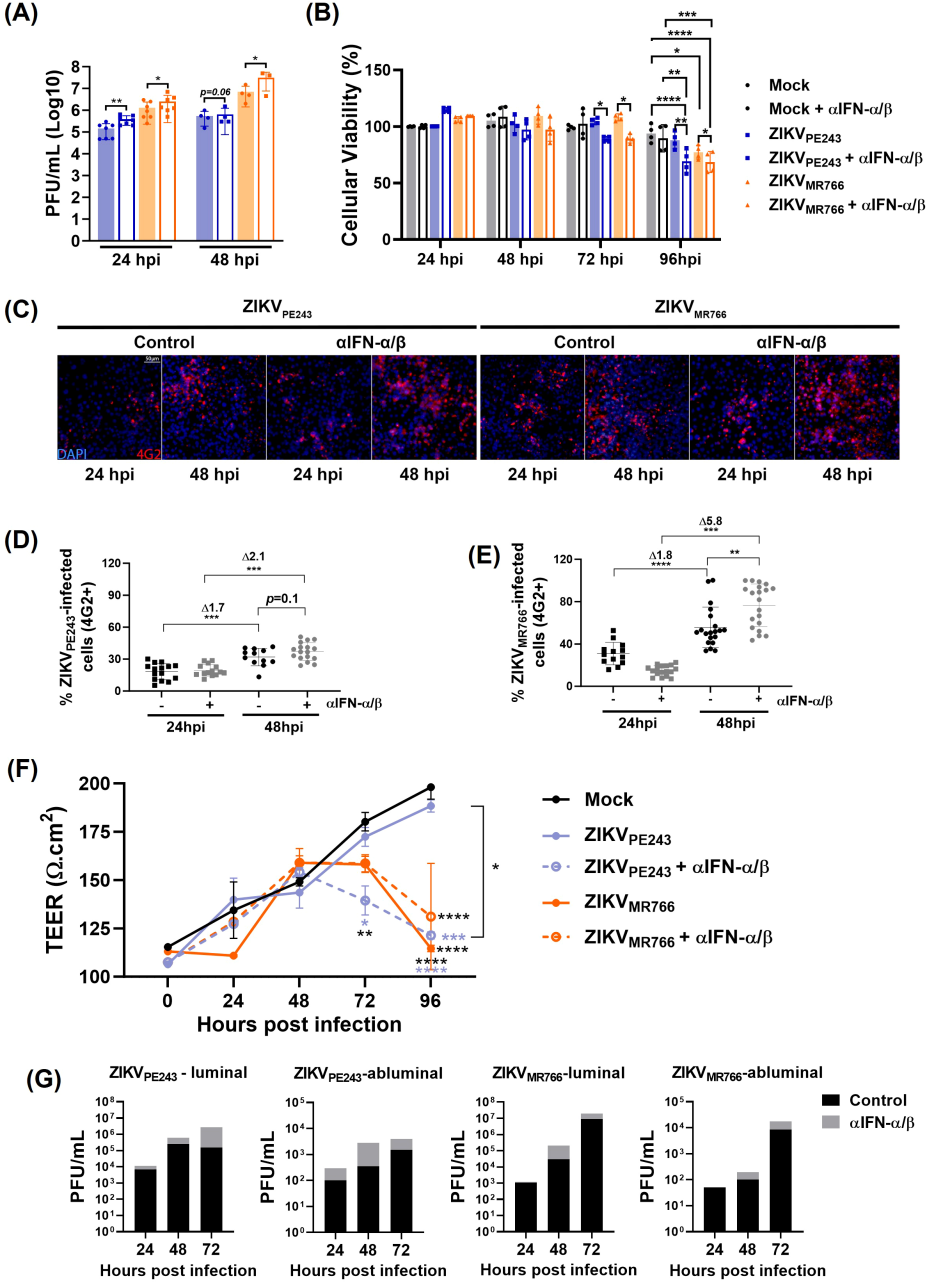
Type I IFN limits ZIKV replication, dissemination and preserves HBMECs barrier integrity. **(A)** HBMECs were infected with ZIKV strains (MOI=0.1) in the presence or absence of a combination of neutralizing anti-IFN-α and anti-IFN-β antibodies (αIFN-α/β). At 24 and 48 hpi, virus replication was evaluated by plaque assay; graph bars represent the mean and SD of four (24 h) and two (48 h) independent assays, and statistical analyses were performed by paired t-test. **(B)** Cells were cultured as in A, and viability was evaluated at the indicated time points using a Cell Titer assay. Data indicates the mean and SD of four independent experiments, and statistical analyses were performed by two-way ANOVA and Tukey’s multiple comparison test. **(C–E)** Cells were cultured as in **(A)** and, at 24 and 48hpi, the cells were stained with 4G2 antibody. Representative figures are shown in **(C)** and the average and SD of the frequency of ZIKV-infected cells (% 4G2+) is depicted in **(D)** and **(E)**. Data was calculated from two experiments, including 20 images. Statistical analyses were performed by one-way ANOVA. **(F, G)** HBMECs were cultured onto transwell plates and the cells were mock-treated or infected with ZIKV_PE243_ or ZIKV_MR766_ and then treated or not with αIFN-α/β. Transendothelial electrical resistance (TEER) were measured in indicated time points **(F)** and viral titer was measured in the luminal and abluminal chambers of the transwell plates by plaque assay **(G)**; statistical analyses were performed by two-way ANOVA, followed by Tukey’s multiple comparison test; *p<0.05; **p<0.005; ***p<0.0005; ****p<0.0001; blue and black stars (*) indicate comparison with ZIKV_PE243_ or mock, respectively.

Using a different experimental setting, a combination of anti-IFN-α and anti-IFN-β neutralizing antibodies was added at the beginning of ZIKV infection to block the effect of endogenous virus-induced IFN. As controls, cell viability was evaluated in antibody-treated cultures, and no significant toxicity was detected from 24 h to 96 h (**Supplementary Figure S3A**). Also, neutralization activity at non-toxic concentration was confirmed by measuring IFN response in HBMEC_ISREluc_ reporter cells treated with recombinant IFN-β or infected with DENV, in the presence of the neutralizing antibodies (**Supplementary Figure S3B**). Analysis of ZIKV-infected cultures revealed that type I IFN neutralization significantly increased virus replication and cell death (**Figures 7A, B**), supporting that endogenous IFN-signaling protects HBMEC from ZIKV-induced cytopathic effect.

The frequency of infected cells in these cultures was monitored through immunofluorescence analyses using the 4G2 antibody. The percentage of HBMECs infected with ZIKV_PE243_ and ZIKV_MR766_ increased by 1.7-fold and 1.8-fold, respectively, between 24 and 48 hours. In contrast, when the cells were infected in the presence of anti-IFN, the fold increases rose to 2 and 5.8, respectively (**Figures 7C-E**), suggesting that IFN neutralization enhanced virus dissemination.

Earlier data from our group showed that ZIKV strains can cross the HBMEC monolayer without causing its disruption (Papa et al., 2017). To investigate whether interferon produced by infected cells acts as an endogenous protective factor, we sought to evaluate its ability to preserve endothelial barrier integrity and to limit, at least partially, viral extravasation across the monolayer. To this end, HBMECs were seeded in a transwell system and transendothelial electric resistance (TEER) was measured for at various time points post infection, in the presence or absence of anti-IFN neutralizing antibodies. As expected, ZIKV_PE243_ infection did not impact HBMEC integrity, however, a significant TEER reduction was detected from 72h.p.i. in the wells treated with anti-IFN (**Figure 7F**). In contrast, ZIKV_MR766_-infected HBMECs showed a reduction in TEER after 72 and 96 h.p.i., which was not affected significantly by IFN neutralization.

Virus titers were assessed in the upper (luminal) and lower (abluminal) chambers. Increased concentration of infectious particles was detected in both compartments of ZIKV_PE243_ and ZIKV_MR766_ cultures, supporting that IFN neutralization facilitated ZIKV dissemination across and through the HBMEC monolayer (**Figure 7G**). These findings highlight the role of type I IFNs in preserving BBB endothelial cells viability, function and protection of against viral dissemination.

### 3.4 IFNAR expression in endothelial cells partially restricts ZIKV infection and disease *in vivo*

**Figure 8:**
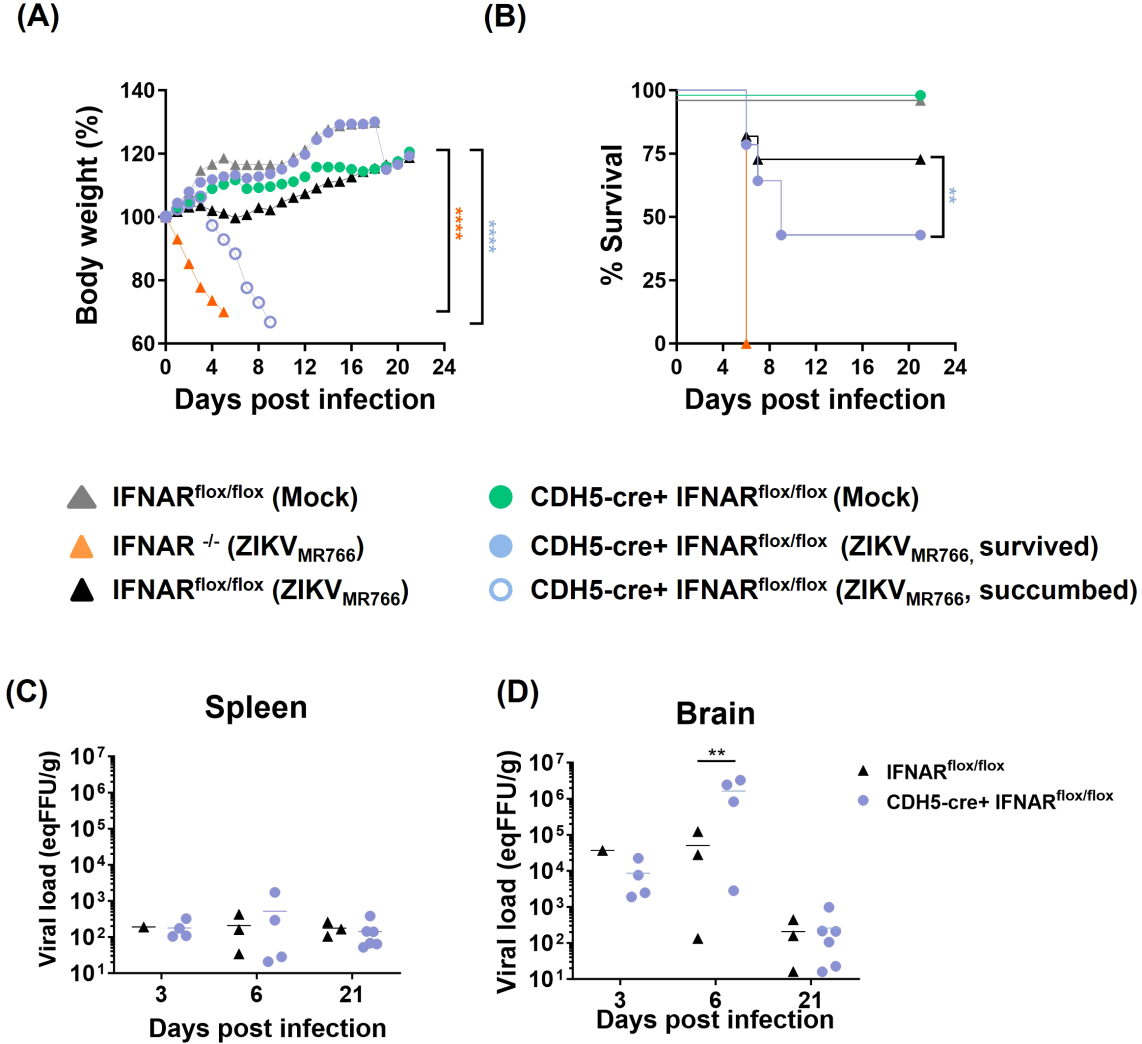
IFNAR deficiency conditioned to endothelial cells partially protects mice from ZIKV-mediated disease. Fully IFNAR-deficient mice (IFNAR-/-) and mice deficient in IFNAR only in endothelial cells (CDH5_cre_ IFNAR^flox/flox^), as well as IFNAR^flox/flox^ (IFNAR^flox/flox^) control mice were intravenously infected with 2x10^5^`PFU of ZIKV_MR766_ or inoculated with mock control. Mice were followed for 21 days. **(A)** Survival curve showed statistical significance (**p<0.01) as evaluated by log-rank (Mantel-Cox) tests. **(B)** Mice body weight was followed until the sacrifice and the results were statistically analyzed using two-way Anova and Tukey’s posttest; ****p<0.0001 in relation to IFNAR^flox/flox^ (mock and ZIKV_MR766_), CDH5-cre+IFNAR^flox/flox^ (mock and ZIKV_MR766_ survived). **(C, D)** Viral titer in the spleens **(C)** and the brains **(D)** were evaluated by plaque assay; statistical analyses were performed at 6 dpi by one-way anova and Tukey’s posttest. *p<0.05; **p<0.01 ***p<0.001; ****p<0.0001.

We had previously demonstrated that 4-week-old IFNAR-deficient SvA129 mice systemically infected (i.v.) with ZIKV_MR766_, but not with ZIKV_PE243_, resulted in increased virus dissemination to the CNS, followed by BBB disruption, development of neurological syndrome, and death (Papa et al., 2017; Lucas et al., 2018). To specifically address the role of endothelium-mediated IFN response, we performed the same infection protocol using a mouse model generated from the mating between IFNAR^flox/flox^ (IFNAR^fl/fl^) and CDH5_cre,_ which express Cre-recombinase under the regulatory control of the VE-Cadherin (Alva et al., 2006), thus restricting IFNAR deficiency to vascular endothelial cells. CDH5_cre+_ IFNAR^fl/fl^ were mock-inoculated or i.v. infected with ZIKV_MR766,_ and mice were followed to assess survival, body weight, and viral load in different tissues. Fully deficient IFNAR^-/-^ and IFNAR^fl/fl^ mice were used as controls. As expected, all mock-infected mice survived infection, whereas 100% of infected IFNAR^-/-^ succumbed at 6 dpi. Interestingly, about 55% of CDH5_cre_+ IFNAR^fl/fl^ mice succumbed to infection or presented an end-stage illness and were euthanized from 6 to 9 dpi (**Figures 8A, B**). Indeed, we found striking differences between CDH5_cre_+ IFNAR^fl/fl^ and control IFNAR^fl/fl^ mice regarding survival (~75% vs ~45% survival, *p*=0.0142) and weight (% starting weight 117% vs 66% at each endpoint; *p*<0.0001) (**Figures 7A, B**). This data demonstrates that IFNAR response in endothelial cells partially protects mice from lethal ZIKV disease.

Finally, viral load in the brains and spleen were then accessed at different time points after infection. No difference was detected at 3 dpi, when all mice groups were clinically stable, with no weight loss nor clinical signs. At 6 dpi, four mice from the CDH5_cre_+ IFNAR^fl/fl^ group were euthanized, being two of them moribund, presenting weight loss above 20% and intense lethargy. Three mice from the control (IFNAR^f/f^) group were also euthanized, for comparison. Increased viral load were detected in three out of four (75%) mice from the CDH5_cre_+ IFNAR^fl/fl^ group, with titers 30–120 times greater than the mouse with the highest viral load from the control group (*p*=0.0072) (**Figures 8C, D**). When moribund and survivors’ mice were independently analyzed, *p* values of 0.02 (CDH5_cre_+ IFNAR^fl/fl^ moribund vs IFNAR^f/f^) and of 0.03 (CDH5_cre_+ IFNAR^fl/fl^ moribund vs CDH5_cre_+ IFNAR^fl/fl^ survivors) were determined. Other mice from CDH5_cre_+ IFNAR^fl/fl^ group were found critically ill at different days post infection and were also analyzed. One mouse was euthanized at 7 dpi and three others at 9 dpi. All those mice presented a higher viral load compared to control mice at 6 dpi (above 5x10^4^ eq PFU/g tissue). At 21 dpi, viral titers decreased in both groups to 10^1–^10^2^ eqPFU/g, and no differences were further detected.

## 4 Discussion

This study demonstrates that two ZIKV strains, ZIKV_PE243_ (Asian/American lineage) and ZIKV_MR766_ (African prototype), induce distinct molecular signatures in HBMEC, an established model of the BBB. Among the pathways modulated, type I IFNs signaling stood out as one of the most marked differences between the strains. This prompted us to further investigate how strain-specific modulation of IFN responses in endothelial cells could influence viral replication, cytotoxicity, and BBB integrity, using an integrated approach that combined transcriptomic, functional, and *in vivo* analyses.

So far, only isolates belonging to the Asian genotype have been associated with congenital and neonatal problems and neurological disorders (Musso et al., 2019; Liu et al., 2019). However, experimental *in vitro* and *in vivo* models have consistently shown that African genotypes isolates may show a more virulent profile than the Asian strains (Anfasa et al., 2017; Bowen et al., 2017; Shao et al., 2017; Tripathi et al., 2017; Smith et al., 2017). These findings align with our previous work showing that ZIKV_MR766_ displays higher replication rates, cytotoxicity, and BBB disruption *in vitro* (Papa et al., 2017), correlating with increased neurological outcomes and mortality *in vivo* (Lucas et al., 2018). Similarly, Aubry et al. (2021) reported that recent African isolates caused higher viremia, morbidity, and fetal death in IFNAR/IFNAGR-deficient mice, compared to pandemic and nonpandemic Asian strains, suggesting that the neuropathogenic potential of first ones might have been underestimated or misdiagnosed.

Our transcriptome analysis showed broader and more pronounced transcriptional changes in ZIKV_MR766_–infected HBMECs, whereas ZIKV_PE243_ induced a profile closer to mock controls, consistent with milder cytopathic effects. Previous studies in ZIKV-infected patients and diverse *in vitro* models have reported heterogeneous activation of IFN-related pathways. Infection of primary macrophages with the Asian strain COL345Si induced upregulation of genes related to RNA modification and post-transcriptional regulation (Fernandez et al., 2022), together with enhanced expression of several ISGs, yet with only a slight induction of type I IFN transcripts. Infection of primary mDCs with another Asian strain (PRVABC59), however, had minimal impact on global transcriptional profile and did not enhance the expression of genes involved in pathogen sensing or interferon signaling (Sun et al., 2017), which closely resemble the profile of HBMECs infected with ZIKV_PE243_. Conversely, mDCs isolated from acutely infected patients showed major alterations in gene expression signatures; while IFN-related genes were downregulated (Sun et al., 2017). Consistent with our findings, these studies suggest that limited activation of sensing and innate immune pathways may represent an evasion strategy of epidemic ZIKV strains, thereby enabling more silent dissemination within individuals and across populations.

The distinct transcriptional modulation IRFs and ISGs led us to identify the upstream sensors responsible for this activation. Selective depletion of RIG-I or TLR3 revealed RIG-I as the primary sensor mediating IFN-β induction upon ZIKV infection, consistent with studies conducted with various human cell types (Hamel et al., 2015; Singh et al., 2017; Hertzog et al., 2018; Schilling et al., 2020; Plociennikowska et al., 2021). Indeed, a conserved stem-loop structure in the 5’ UTR region in the flaviviruses genome has been proposed as a RIG-I agonist (Chazal et al., 2018), supporting the role of RIG-I as a common viral RNA sensor during infection with both ZIKV strains. MDA5 expression was also upregulated in ZIKV_MR766_-infected cells, suggesting a possible contribution to IFN-β induction. However, since RIG-I and MDA5 share overlapping functions and are both IFN-inducible, this increase could reflect secondary signaling. Furthermore, RIG-I depletion nearly abolished IFN-β production, underscoring its predominant role in initiating antiviral signaling in our system.

ZIKV_MR766_ induced a more rapid and robust IFN-β response compared with ZIKV_PE243_, but it could not be attribute to higher viral RNA levels at early time points. Inhibition of IFN production by ZIKV nonstructural (NS) proteins was previously described (Bowen et al., 2017; Hertzog et al., 2018; Fanunza et al., 2021a, b), and may be triggered by several pathways, including interaction with RIG-I CARD domain, TBK1 or IRF3, hampering the activation of TBK1/IRF3 signaling (Wu et al., 2017; Xia et al., 2018; Lin et al., 2019; Li et al., 2020). Also, interaction of the viral structural capsid protein with TRIM25 has been shown to regulate RIG-I ubiquitination and downstream IFN production (Airo et al., 2022). We identified few substitutions in the capsid sequence, including the K101R, which was associated with enhanced neurovirulence of ZIKV African strains (Song et al., 2023), suggesting that strain-specific differences in capsid composition or structure may contribute to RIG-I activation and distinct IFN-β expression kinetics.

Despite efficient IFN-β production, both ZIKV strains productively replicated in HBMECs, indicating virus evasion from IFN-mediated antiviral signaling. Flaviviruses can counteract type I IFN response through multiple pathways. A major described mechanism involves STAT2 ubiquitination and proteasomal degradation by the NS5 protein, previously observed in DENV and ZIKV infections (Morrison et al., 2013; Grant et al., 2016). In ZIKV, STAT2 degradation depends on its interaction with NS5 RNA-dependent RNA polymerase (RdRP) domain (Grant et al., 2016; Wang et al., 2020; Parisien et al., 2022), and may be accelerated by downmodulation of *de novo* host proteins synthesis (Shu et al., 2021). Another important mechanism relies on inhibition of STAT1 or STAT2 phosphorylation by NS proteins, reported in DENV, WNV, Japanese encephalitis virus (JEV), and Tick-born encephalitis virus (TBEV) (Muñoz-Jordan et al., 2003, Lin et al., 2006; Laurent-Rolle et al., 2010; Yang et al., 2020). However, IFN evasion is not equally detected in all cell types (Yang et al., 2021).

In HBMECs, we observed a remarkable reduction of STAT1 phosphorylation and almost suppression of STAT2 expression, indicating these as major mechanisms hampering IFN-β signal transduction. Those events were even more pronounced in the cells infected with ZIKV_MR766_ in relation to ZIKV_PE243_. A variable magnitude of IFN signaling inhibition has also been reported following NS5 transfection or infection with distinct ZIKV strains (Grant et al., 2016; Xia et al., 2018). Although we have identified amino acid substitutions in the NS5 sequences of ZIKV_MR766_ and ZIKV_PE243_, none correspond to residues previously associated with RNA synthesis efficiency or with STAT2 interaction and inhibition (Zhao et al., 2017; Wang et al., 2020; Conde et al., 2020), and remains to be further explored.

ZIKV also produces 3’UTR derived sfRNA (Akiyama et al., 2016; Donald et al., 2016), which has been associated with cytopathic effect and modulation of antiviral responses in multiple flavivirus infection models (Pijlman et al., 2008; Schuessler et al., 2012). Transfection of A549 cells with a plasmid containing the sfRNA of ZIKV_PE243_ inhibited poly I:C-induced IFN response, mostly due to antagonizing RIG-I signaling (Donald et al., 2016). A comparison of different ZIKV isolates, including the ones investigated here, showed that despite nucleotide differences in the 3′ UTRs, these are unlikely to affect the predicted secondary structures responsible for sfRNA, suggesting conserved functions in antagonizing type I interferon responses.

Importantly, although HBMECs infected with ZIKV resist the effects mediated by type I IFN, IFN neutralization in low MOI infected cultures led to increased viral load, cytotoxicity and virus dissemination. In agreement with our previous findings (Papa et al., 2017) and others (Wang et al., 2023), ZIKV_PE243_ infection alone did not affect endothelial permeability throughout the experiment. However, IFN neutralization rendered ZIKV_PE243_-infected monolayers permeable, indicating that the preserved integrity observed under normal conditions depends on type I IFN signaling. Differently from earlier studies (Papa et al., 2017; Wang et al., 2023), where TEER values remained stable across the entire culture period, here we observed an initial increase in TEER during the early phase of the assay. This is likely due to the monolayers starting slightly below full confluence, as a lower MOI was applied in these conditions. Despite this initial rise, TEER values were not influenced by infection up to 48 h. Thereafter, however, neutralization of IFN led to a gradual decline in TEER, more clearly evident in ZIKV_PE243_-infected cultures, where barrier integrity is otherwise preserved.

Also, addition of IFN-β 24 h before infection inhibited virus replication. IFN-β IC_50_ and IC_90_ were significantly higher in the cultures infected with ZIKV_MR766_ in relation to the ones infected with ZIKV_PE423_, indicating greater resistance to IFN-mediated antiviral activity, consistent with higher virus titers later upon infection. Accordingly, pretreating fibroblasts with type I IFN also reduced ZIKV replication (Hamel et al., 2015), and increased resistance of ZIKV_MR766_ in comparison with the Asian strain PRBAVC59, had been previously reported in A549 cells (Gobillot et al., 2020).

Recent findings by Lefevre et al. (2024) identified lineage-specific upstream open reading frames (uORFs) in the 5′ untranslated region (UTR) of ZIKV that may modulate viral replication and tropism. In African isolates, a single uORF is present, whereas in Asian/American strain, including ZIKV_PE243_, a nucleotide insertion at position 81 divides this uORF into two distinct uORFs - uORF1 and uORF2. Using human brain organoids and glioblastoma U251 cells, the authors showed that viruses bearing the African-like uORF or lacking uORF1 reached higher titers than the American wild-type strain. While direct evidence was not demonstrated, the data suggest that the African-like uORF arrangement may confer resistance to eIF2α-mediated translational arrest, potentially favoring replication under IFN-induced stress. Our genomic analysis confirmed the presence of nt81 insertion in ZIKV_PE243_, but not in ZIKV_MR766_, possibly implicating lineage-specific uORF as an additional mechanism of IFN evasion. In fact, our comparative genomic analysis identified amino acid changes in the 5′ UTR, capsid, and NS5 regions of ZIKV_PE243_ and ZIKV_MR766_, including ones previously associated with differences in viral biology, as well as novel substitutions whose functional relevance remains unknown. While reverse-genetic or gene-editing approaches will be required to define their precise roles, it is plausible that sequence variations across these genomic regions act synergistically to modulate IFN signaling, viral replication, and disease outcome, thereby shaping strain-specific virulence.

Evasion of type I IFN signaling has been linked to epidemiological fitness and disease severity during flavivirus infections, including ZIKV (Manokaran et al., 2015; Castro-Jimenez et al., 2022). To specifically evaluate the contribution of endothelial cells in these processes, we employed an *in vivo* model in which IFNAR deficiency is restricted to the endothelium. Our previous studies showed that intravenous infection of IFNAR-deficient mice with ZIKV_MR766_, but not ZIKV_PE243_, led to BBB disruption and lethality (Lucas et al., 2018). Using identical infection parameters (4-week-old mice; 2×10^5^ PFU) for direct comparison, we now demonstrated, for the first time, that endothelial-specific IFNAR depletion also rendered mice susceptible to ZIKV_MR766_-induced lethality, accompanied by greater neuroinvasiveness.

In an *in vivo* context, however, other components of the neurovascular unit might play an additional role in regulating BBB integrity and antiviral response. Previous studies have reported that pericytes, astrocytes and microglial cells are susceptible to be ZIKV, potentially contributing to IFN-β-mediated protection (Chen et al., 2018; Kim et al., 2020; Stefanik et al., 2018; Ojha et al., 2019). Astrocytes have been recently identified as innate immune sentinel cells capable of sensing ZIKV and producing IFN-β in human fetal brain explants and iPSC-derived neural cultures (Stokes et al., 2025), and infection of astrocytic endfeet has been associated with BBB disruption in IFNAR-deficient mice (Jurado et al., 2018). However, in contrast to our observations in HBMECs, infection of human astrocytes with Asian ZIKV strains resulted in higher infectivity and cell death than with African isolates (Ojha et al., 2019). Also, transcriptome analysis of primary astrocytes infected with ZIKV_PE243_ showed downregulation of IFN-signaling pathways (Geddes et al., 2021), in accordance with the observed profile in HBMECs. Microglia are also recognized targets of ZIKV infection, and interferon responses influence viral replication dynamics (Hanrath et al., 2022). Lineage-specific differences have been reported in this cell type, although the patterns diverge between human and mouse microglial models. Asian strains consistently elicit weaker inflammatory activation than African isolates in both species; however, they replicate more efficiently in human microglia (Bird et al., 2024), but not in mouse microglia (Oliveira et al., 2023). In pericyte cultures, neutralization of type I IFNs increased viral replication at later time points, suggesting a protective antiviral effect (Kim et al., 2020).

Still, in our model, other BBB-associated cells were not included in the *in vitro* setting; therefore, IFN neutralization primarily blocked autocrine signaling in endothelial cells. Furthermore, although IFNAR deficiency *in vivo* was not restricted to endothelial cells from the BBB, these findings collectively reinforce the importance of endothelial IFN signaling in maintaining barrier integrity and controlling ZIKV neuroinvasiveness and lethality, even though direct evidence linking viral replication in these cells remains to be established.

Other *in vitro* models have also supported a protective role for endothelial IFN responses in restricting viral replication and neuroinvasion. Using iPSC-derived human BMECs infected with a panel of alphaviruses and flaviviruses, Cheng et al. (2022) showed an inverse correlation between IFITM1 expression and both viral replication and the ability to cross the iBMEC monolayer. Similarly, BMEC derived from IFNAR-deficient mice infected with WNV exhibit reduced IFN and increased inflammatory cytokines, leading to barrier dysfunction (Daniels et al., 2014). Co-culture experiments with astrocytes further demonstrated that endothelial IFN signaling played a predominant role in preventing BBB breakdown. Therefore, studies using IFNAR-deficient HBMECs will contribute of dissect the impact of endothelial IFN signaling to ZIKV replication, metabolic modulation, and BBB integrity.

In summary, our findings demonstrate that type I IFN signaling in brain endothelial cells is a key determinant of BBB integrity and resistance to ZIKV infection. By inducing classical antiviral response, endothelial IFN limit viral replication, cytotoxicity, and barrier permeability, thereby contributing to disease resistance. However, this protection is only partial, since ZIKV proteins interfere with IFN signaling, rendering infected cells less responsive to its antiviral effects. The two viral strains analyzed exhibited distinct biological profiles: ZIKVMR766 induced stronger IFN and inflammatory responses yet showed greater resistance to IFN-mediated restriction, correlating with enhanced BBB disruption and disease severity *in vivo*.

Although BBB dysfunction is a hallmark of several viral infections associated with neurological disease (Mustafa et al., 2019; Greene et al., 2024; Coelho et al., 2025; Boardman et al., 2025), the specific contribution of endothelial cells and their interferon-mediated responses remains poorly understood. As the structural and immunological core of the BBB, endothelial cells actively coordinate antiviral defense, inflammatory regulation, and barrier preservation. Our study thus reinforces their role as central players in CNS protection. Beyond ZIKV, these findings provide a conceptual framework for investigating IFN-dependent mechanisms of BBB protection and neuroinvasion across other neurotropic viral infections, guiding future development of therapeutic strategies aiming to maintain endothelial integrity and mitigate virus-induced neurological disorders.

## Data Availability

The libraries sequenced in our study are available at SRA (www.ncbi.nlm.nih.gov/sra) under BioProject accession number PRJNA1337481 and accession numbers SAMN52321194–SAMN52321202.
