# Correction: Distinct ZIKV strain signatures and type I IFN modulation reveal a protective role of brain endothelial interferon signaling *in vitro* and *in vivo*

**DOI:** 10.3389/fcimb.2025.1762181

**Published:** 2025-12-16

**Authors:** Luan Rocha Lima, Yasmin Mucunã Mustafá, Paula Luize Camargos Fonseca, Sharton Vinícius Antunes Coelho, Pierina Lorencini Parisi, Camila Lopes Simeoni, Lana Monteiro Meuren, Bruno Braz Bezerra, Nathane Cunha Mebus-Antunes, Flavio Matassoli, Jose Luiz Proença-Modena, Renato Santana Aguiar, Luciana Barros de Arruda

**Affiliations:** 1 Departamento de Virologia, Instituto de Microbiologia Paulo de Góes, Universidade Federal do Rio de Janeiro (UFRJ), Rio de Janeiro, RJ, Brazil; 2 Departamento de Genética, Ecologia e Evolução, Instituto de Ciências Biológicas, Universidade Federal de Minas Gerais, Belo Horizonte, Brazil; 3 Departamento de Genética, Microbiologia e Imunologia, Instituto de Biologia, Universidade Estadual de Campinas (UNICAMP), Campinas, SP, Brazil; 4 Instituto de Bioquímica Médica Leopoldo De Meis (IBqM), Universidade Federal do Rio de Janeiro (UFRJ), Rio de Janeiro, RJ, Brazil; 5 Laboratory of Immunoregulation, National Institute of Allergy and Infectious Diseases (NIAID), National Institutes of Health, Bethesda, MD, United States; 6 Instituto D’OR de Pesquisa e Ensino, Rio de Janeiro, Rio de Janeiro, Brazil

**Keywords:** Zika virus, interferon, endothelial cells, blood brain barrier, neuroinvasion

There was a mistake in **Figure 5** as published. An arrow indicating a modification in the 5’UTR was missing in **Figures 5A, B**, and one of the colors did not match the legend in **Figure 5E**. The corrected **Figure 5** appears below.

**Figure 5:**
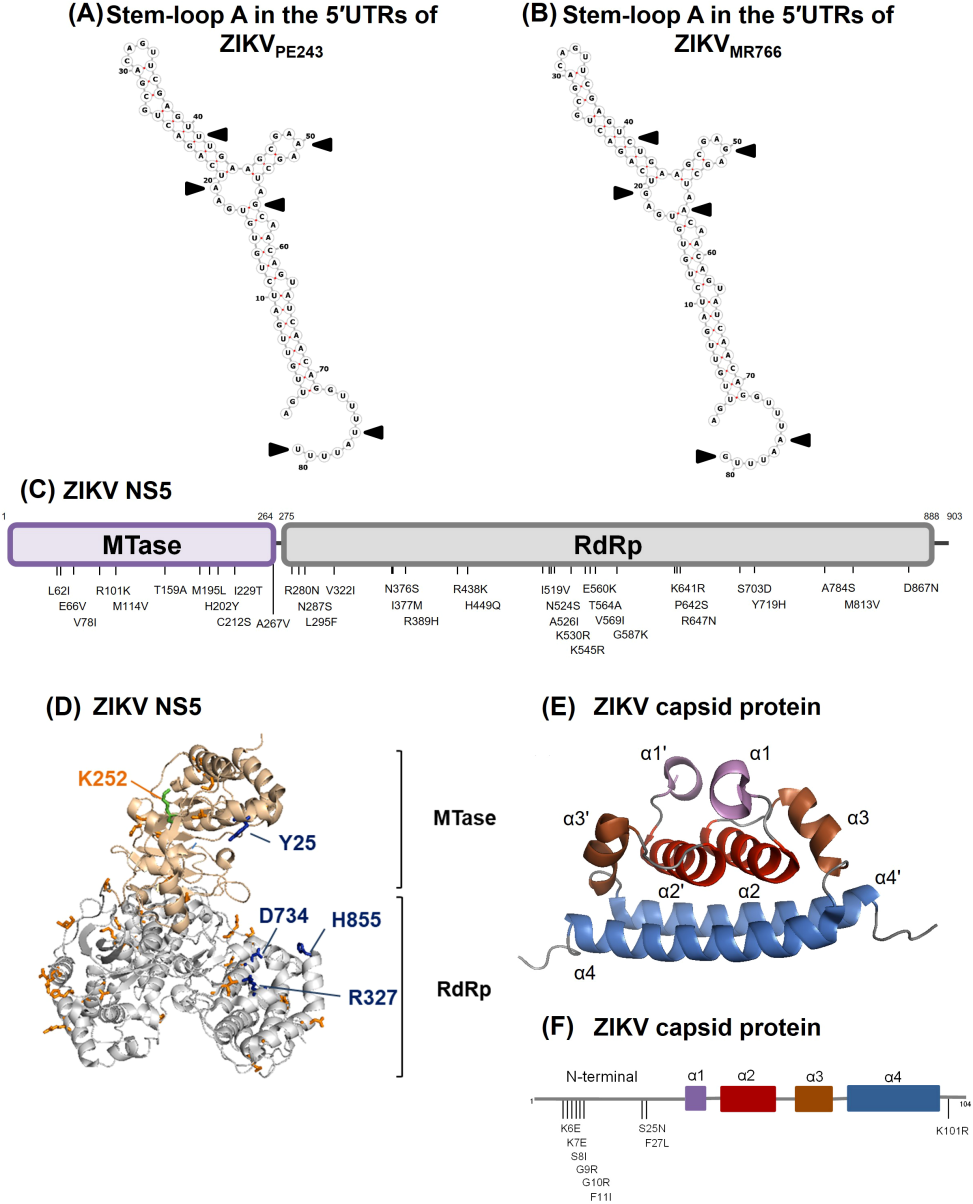
Comparison of genomic and amino acid sequences by the 5’UTR, NS5 and capsid protein from ZIKV_MR766_ (NC_012532.1) and ZIKV_PE243_ (GenBank KX197192.1). **(A, B)** Sequence and predicted secondary structure of stem-loop A in the 5′UTRs from ZIKV_PE243_**(A)** and ZIKV_MR766_**(B)**. The black arrows indicate sequence variations in the 5′-UTR. **(C)** Schematic representation of ZIKV NS5 from ZIKV_MR766_ indicating the amino acid substitutions in the NS5 from ZIKV_PE243_. MTase and RdRp domains are colored purple and gray, respectively. **(D)** Ribbon representation showing the MTase (light pink) and RdRp (gray) domains of ZIKV_MR766_ NS5. The locations of residues that differed in NS5 from ZIKV_PE243_ in the context of ZIKV_MR766_ NS5 structure (PDB, 5U0B) are shown in orange sticks. The residues Y25, R327, D734 and H855 are highlighted in blue sticks, and the K252 residue (green stick) is labeled in orange. **(E)** Ribbon representation showing the structure of the ZIKV capsid protein (PDB, 6C44), with the α-helix pairs indicated: α1/α1’ (purple), α2/α2’ (red), α3/α3’ (brown), and α4/α4’ (blue). **(F)** Schematic representation of the capsid protein from ZIKV_PE243_ indicating the amino acid substitutions from ZIKV_MR766_. The α-helices are colored according to protein structure in **(E)**.

The original version of this article has been updated.

