# Supplementary material for: Distinct ZIKV strain signatures and type I IFN modulation reveal a protective role of brain endothelial interferon signaling in vitro and in vivo
Source: Front Cell Infect Microbiol. 2025 Dec 3;15:1726007. doi: 10.3389/fcimb.2025.1726007 (PMC12752121; doi:10.3389/fcimb.2025.1726007)
Supplement: Supplementary file 1 [file DataSheet1.pdf]

Table S1: Shared and unique upregulated genes

PE243	MR766	Gene	Gene_ID	Gene_Name	Shared_Conditions	Condition_Count	Category
1	1	ENSG00000065518.7	ENSG00000065518	NDUFB4	PE243, MR766	2	Shared
1	0	ENSG00000068976.13	ENSG00000068976	PYGM	PE243	1	Unique
1	0	ENSG00000100353.17	ENSG00000100353	EIF3D	PE243	1	Unique
1	0	ENSG00000104549.11	ENSG00000104549	SQLE	PE243	1	Unique
1	0	ENSG00000105810.9	ENSG00000105810	CDK6	PE243	1	Unique
1	1	ENSG00000105825.11	ENSG00000105825	TFPI2	PE243, MR766	2	Shared
1	0	ENSG00000107959.15	ENSG00000107959	PITRM1	PE243	1	Unique
1	0	ENSG00000114315.3	ENSG00000114315	HES1	PE243	1	Unique
1	0	ENSG00000115053.15	ENSG00000115053	NCL	PE243	1	Unique
1	1	ENSG00000115267.5	ENSG00000115267	IFIH1	PE243, MR766	2	Shared
1	0	ENSG00000116641.17	ENSG00000116641	DOCK7	PE243	1	Unique
1	0	ENSG00000116741.7	ENSG00000116741	RGS2	PE243	1	Unique
1	0	ENSG00000116962.14	ENSG00000116962	NID1	PE243	1	Unique
1	1	ENSG00000118193.11	ENSG00000118193	KIF14	PE243, MR766	2	Shared
1	1	ENSG00000118680.12	ENSG00000118680	MYL12B	PE243, MR766	2	Shared
1	1	ENSG00000125740.13	ENSG00000125740	FOSB	PE243, MR766	2	Shared
1	0	ENSG00000131747.14	ENSG00000131747	TOP2A	PE243	1	Unique
1	0	ENSG00000133059.16	ENSG00000133059	DSTYK	PE243	1	Unique
1	1	ENSG00000133065.10	ENSG00000133065	SLC41A1	PE243, MR766	2	Shared
1	1	ENSG00000141543.9	ENSG00000141543	EIF4A3	PE243, MR766	2	Shared
1	0	ENSG00000145293.15	ENSG00000145293	ENOPH1	PE243	1	Unique
1	1	ENSG00000146112.11	ENSG00000146112	PPP1R18	PE243, MR766	2	Shared
1	0	ENSG00000157077.14	ENSG00000157077	ZFYVE9	PE243	1	Unique
1	0	ENSG00000162607.12	ENSG00000162607	USP1	PE243	1	Unique
1	1	ENSG00000163041.9	ENSG00000163041	H3-3A	PE243, MR766	2	Shared
1	1	ENSG00000163050.16	ENSG00000163050	COQ8A	PE243, MR766	2	Shared
1	0	ENSG00000167378.8	ENSG00000167378	IRGQ	PE243	1	Unique
1	1	ENSG00000167658.15	ENSG00000167658	EEF2	PE243, MR766	2	Shared
1	0	ENSG00000168672.3	ENSG00000168672	LRATD2	PE243	1	Unique
1	1	ENSG00000170345.9	ENSG00000170345	FOS	PE243, MR766	2	Shared
1	1	ENSG00000170779.10	ENSG00000170779	CDCA4	PE243, MR766	2	Shared
1	1	ENSG00000175197.11	ENSG00000175197	DDIT3	PE243, MR766	2	Shared
1	1	ENSG00000185745.9	ENSG00000185745	IFIT1	PE243, MR766	2	Shared
1	0	ENSG00000186272.12	ENSG00000186272	ZNF17	PE243	1	Unique
1	0	ENSG00000186594.14	ENSG00000186594	MIR22HG	PE243	1	Unique
1	0	ENSG00000201183.1	ENSG00000201183	RNVU1-3	PE243	1	Unique
1	0	ENSG00000213079.9	ENSG00000213079	SCAF8	PE243	1	Unique
1	1	ENSG00000260772.1	ENSG00000260772		PE243, MR766	2	Shared
1	0	ENSG00000261324.2	ENSG00000261324		PE243	1	Unique
1	0	ENSG00000261799.1	ENSG00000261799		PE243	1	Unique
1	0	ENSG00000264112.1	ENSG00000264112		PE243	1	Unique
1	0	ENSG00000275318.1	ENSG00000275318		PE243	1	Unique
1	0	ENSG00000277151.1	ENSG00000277151		PE243	1	Unique

1	1	ENSG00000277610.1	ENSG00000277610	RNVU1-4	PE243, MR766	2	Shared
0	1	ENSG00000005812.10	ENSG00000005812	FBXL3	MR766	1	Unique
0	1	ENSG00000006015.17	ENSG00000006015	REX1BD	MR766	1	Unique
0	1	ENSG00000007376.7	ENSG00000007376	RPUSD1	MR766	1	Unique
0	1	ENSG00000010818.9	ENSG00000010818	HIVEP2	MR766	1	Unique
0	1	ENSG00000011638.10	ENSG00000011638	LDAF1	MR766	1	Unique
0	1	ENSG00000019485.13	ENSG00000019485	PRDM11	MR766	1	Unique
0	1	ENSG00000034677.12	ENSG00000034677	RNF19A	MR766	1	Unique
0	1	ENSG00000042753.11	ENSG00000042753	AP2S1	MR766	1	Unique
0	1	ENSG00000063177.12	ENSG00000063177	RPL18	MR766	1	Unique
0	1	ENSG00000064490.13	ENSG00000064490	RFXANK	MR766	1	Unique
0	1	ENSG00000068079.7	ENSG00000068079	IFI35	MR766	1	Unique
0	1	ENSG00000070444.14	ENSG00000070444	MNT	MR766	1	Unique
0	1	ENSG00000086827.8	ENSG00000086827	ZW10	MR766	1	Unique
0	1	ENSG00000100345.20	ENSG00000100345	MYH9	MR766	1	Unique
0	1	ENSG00000104805.15	ENSG00000104805	NUCB1	MR766	1	Unique
0	1	ENSG00000106268.15	ENSG00000106268	NUDT1	MR766	1	Unique
0	1	ENSG00000107175.11	ENSG00000107175	CREB3	MR766	1	Unique
0	1	ENSG00000110107.8	ENSG00000110107	PRPF19	MR766	1	Unique
0	1	ENSG00000110108.9	ENSG00000110108	TMEM109	MR766	1	Unique
0	1	ENSG00000111678.10	ENSG00000111678	C12orf57	MR766	1	Unique
0	1	ENSG00000118503.14	ENSG00000118503	TNFAIP3	MR766	1	Unique
0	1	ENSG00000119917.13	ENSG00000119917	IFIT3	MR766	1	Unique
0	1	ENSG00000123091.4	ENSG00000123091	RNF11	MR766	1	Unique
0	1	ENSG00000125347.13	ENSG00000125347	IRF1	MR766	1	Unique
0	1	ENSG00000125703.14	ENSG00000125703	ATG4C	MR766	1	Unique
0	1	ENSG00000126391.13	ENSG00000126391	FRMD8	MR766	1	Unique
0	1	ENSG00000129515.18	ENSG00000129515	SNX6	MR766	1	Unique
0	1	ENSG00000130312.6	ENSG00000130312	MRPL34	MR766	1	Unique
0	1	ENSG00000130589.16	ENSG00000130589	HELZ2	MR766	1	Unique
0	1	ENSG00000131979.18	ENSG00000131979	GCH1	MR766	1	Unique
0	1	ENSG00000135114.12	ENSG00000135114	OASL	MR766	1	Unique
0	1	ENSG00000135632.11	ENSG00000135632	SMYD5	MR766	1	Unique
0	1	ENSG00000135899.17	ENSG00000135899	SP110	MR766	1	Unique
0	1	ENSG00000136143.14	ENSG00000136143	SUCLA2	MR766	1	Unique
0	1	ENSG00000136295.14	ENSG00000136295	TTYH3	MR766	1	Unique
0	1	ENSG00000136628.17	ENSG00000136628	EPRS1	MR766	1	Unique
0	1	ENSG00000136854.19	ENSG00000136854	STXBP1	MR766	1	Unique
0	1	ENSG00000137818.11	ENSG00000137818	RPLP1	MR766	1	Unique
0	1	ENSG00000137824.15	ENSG00000137824	RMDN3	MR766	1	Unique
0	1	ENSG00000138081.20	ENSG00000138081	FBXO11	MR766	1	Unique
0	1	ENSG00000139154.14	ENSG00000139154	AEBP2	MR766	1	Unique
0	1	ENSG00000140006.11	ENSG00000140006	WDR89	MR766	1	Unique
0	1	ENSG00000140320.11	ENSG00000140320	BAHD1	MR766	1	Unique
0	1	ENSG00000141577.13	ENSG00000141577	CEP131	MR766	1	Unique
0	1	ENSG00000142733.14	ENSG00000142733	MAP3K6	MR766	1	Unique

0	1	ENSG00000142910.15	ENSG00000142910	TINAGL1	MR766	1	Unique
0	1	ENSG00000146242.8	ENSG00000146242	TPBG	MR766	1	Unique
0	1	ENSG00000149925.17	ENSG00000149925	ALDOA	MR766	1	Unique
0	1	ENSG00000153487.12	ENSG00000153487	ING1	MR766	1	Unique
0	1	ENSG00000154914.16	ENSG00000154914	USP43	MR766	1	Unique
0	1	ENSG00000155380.11	ENSG00000155380	SLC16A1	MR766	1	Unique
0	1	ENSG00000155827.11	ENSG00000155827	RNF20	MR766	1	Unique
0	1	ENSG00000158406.4	ENSG00000158406	H4C8	MR766	1	Unique
0	1	ENSG00000160877.5	ENSG00000160877	NACC1	MR766	1	Unique
0	1	ENSG00000161057.11	ENSG00000161057	PSMC2	MR766	1	Unique
0	1	ENSG00000162191.13	ENSG00000162191	UBXN1	MR766	1	Unique
0	1	ENSG00000162772.16	ENSG00000162772	ATF3	MR766	1	Unique
0	1	ENSG00000163479.13	ENSG00000163479	SSR2	MR766	1	Unique
0	1	ENSG00000165195.15	ENSG00000165195	PIGA	MR766	1	Unique
0	1	ENSG00000165704.14	ENSG00000165704	HPRT1	MR766	1	Unique
0	1	ENSG00000166454.9	ENSG00000166454	ATMIN	MR766	1	Unique
0	1	ENSG00000172059.10	ENSG00000172059	KLF11	MR766	1	Unique
0	1	ENSG00000172613.7	ENSG00000172613	RAD9A	MR766	1	Unique
0	1	ENSG00000173193.13	ENSG00000173193	PARP14	MR766	1	Unique
0	1	ENSG00000173327.7	ENSG00000173327	MAP3K11	MR766	1	Unique
0	1	ENSG00000174748.18	ENSG00000174748	RPL15	MR766	1	Unique
0	1	ENSG00000177600.8	ENSG00000177600	RPLP2	MR766	1	Unique
0	1	ENSG00000178057.14	ENSG00000178057	NDUFAF3	MR766	1	Unique
0	1	ENSG00000182704.7	ENSG00000182704	TSKU	MR766	1	Unique
0	1	ENSG00000184110.14	ENSG00000184110	EIF3C	MR766	1	Unique
0	1	ENSG00000185022.11	ENSG00000185022	MAFF	MR766	1	Unique
0	1	ENSG00000185507.19	ENSG00000185507	IRF7	MR766	1	Unique
0	1	ENSG00000185896.10	ENSG00000185896	LAMP1	MR766	1	Unique
0	1	ENSG00000197110.8	ENSG00000197110	IFNL3	MR766	1	Unique
0	1	ENSG00000197457.9	ENSG00000197457	STMN3	MR766	1	Unique
0	1	ENSG00000198853.11	ENSG00000198853	RUSC2	MR766	1	Unique
0	1	ENSG00000213860.4	ENSG00000213860	RPL21P75	MR766	1	Unique
0	1	ENSG00000213928.8	ENSG00000213928	IRF9	MR766	1	Unique
0	1	ENSG00000214253.8	ENSG00000214253	FIS1	MR766	1	Unique
0	1	ENSG00000225339.3	ENSG00000225339		MR766	1	Unique
0	1	ENSG00000232956.8	ENSG00000232956	SNHG15	MR766	1	Unique
0	1	ENSG00000235288.3	ENSG00000235288		MR766	1	Unique
0	1	ENSG00000239002.3	ENSG00000239002	SCARNA10	MR766	1	Unique
0	1	ENSG00000241506.1	ENSG00000241506	PSMC1P1	MR766	1	Unique
0	1	ENSG00000242615.1	ENSG00000242615	RPL17P47	MR766	1	Unique
0	1	ENSG00000244038.9	ENSG00000244038	DDOST	MR766	1	Unique
0	1	ENSG00000255302.4	ENSG00000255302	EID1	MR766	1	Unique
0	1	ENSG00000259001.3	ENSG00000259001		MR766	1	Unique
0	1	ENSG00000266412.5	ENSG00000266412	NCOA4	MR766	1	Unique
0	1	ENSG00000269900.3	ENSG00000269900	NA	MR766	1	Unique
0	1	ENSG00000273199.1	ENSG00000273199		MR766	1	Unique

0	1	ENSG00000277947.1	ENSG00000277947	SNORD3D	MR766	1	Unique
0	1	ENSG00000278233.1	ENSG00000278233	RNA5-8SN2	MR766	1	Unique
0	1	ENSG00000279602.1	ENSG00000279602		MR766	1	Unique
