# Supplementary material for: Distinct ZIKV strain signatures and type I IFN modulation reveal a protective role of brain endothelial interferon signaling in vitro and in vivo
Source: Front Cell Infect Microbiol. 2025 Dec 3;15:1726007. doi: 10.3389/fcimb.2025.1726007 (PMC12752121; doi:10.3389/fcimb.2025.1726007)
Supplement: Supplementary file 2 [file DataSheet2.pdf]

Table S2: Shared and unique downregulated genes

PE243	MR766	Gene	Gene_ID	Gene_Name	Shared_Conditions	Condition_Count	Category
1	0	ENSG00000006831.9	ENSG00000006831	ADIPOR2	PE243	1	Unique
1	0	ENSG00000013588.7	ENSG00000013588	GPRC5A	PE243	1	Unique
1	0	ENSG00000034713.7	ENSG00000034713	GABARAPL2	PE243	1	Unique
1	1	ENSG00000061794.12	ENSG00000061794	MRPS35	PE243, MR766	2	Shared
1	1	ENSG00000065060.16	ENSG00000065060	BLTP3A	PE243, MR766	2	Shared
1	0	ENSG00000067955.13	ENSG00000067955	CBFB	PE243	1	Unique
1	0	ENSG00000076356.6	ENSG00000076356	PLXNA2	PE243	1	Unique
1	0	ENSG00000089486.16	ENSG00000089486	CDIP1	PE243	1	Unique
1	0	ENSG00000099899.14	ENSG00000099899	TRMT2A	PE243	1	Unique
1	0	ENSG00000100300.17	ENSG00000100300	TSPO	PE243	1	Unique
1	0	ENSG00000100360.14	ENSG00000100360	IFT27	PE243	1	Unique
1	0	ENSG00000100994.11	ENSG00000100994	PYGB	PE243	1	Unique
1	0	ENSG00000103245.13	ENSG00000103245	CIAO3	PE243	1	Unique
1	0	ENSG00000104518.10	ENSG00000104518	GSDMD	PE243	1	Unique
1	0	ENSG00000105298.13	ENSG00000105298	CACTIN	PE243	1	Unique
1	1	ENSG00000105887.10	ENSG00000105887	MTPN	PE243, MR766	2	Shared
1	0	ENSG00000108262.15	ENSG00000108262	GIT1	PE243	1	Unique
1	0	ENSG00000110881.11	ENSG00000110881	ASIC1	PE243	1	Unique
1	0	ENSG00000113657.12	ENSG00000113657	DPYSL3	PE243	1	Unique
1	0	ENSG00000115163.14	ENSG00000115163	CENPA	PE243	1	Unique
1	0	ENSG00000115355.15	ENSG00000115355	CCDC88A	PE243	1	Unique
1	0	ENSG00000117877.10	ENSG00000117877	POLR1G	PE243	1	Unique
1	0	ENSG00000119408.16	ENSG00000119408	NEK6	PE243	1	Unique
1	0	ENSG00000122507.20	ENSG00000122507	BBS9	PE243	1	Unique
1	0	ENSG00000123091.4	ENSG00000123091	RNF11	PE243	1	Unique
1	0	ENSG00000123870.10	ENSG00000123870	ZNF137P	PE243	1	Unique
1	0	ENSG00000125967.16	ENSG00000125967	NECAB3	PE243	1	Unique
1	1	ENSG00000127337.6	ENSG00000127337	YEATS4	PE243, MR766	2	Shared
1	0	ENSG00000127586.16	ENSG00000127586	CHTF18	PE243	1	Unique
1	0	ENSG00000128791.11	ENSG00000128791	TWSG1	PE243	1	Unique
1	1	ENSG00000129347.19	ENSG00000129347	KRI1	PE243, MR766	2	Shared
1	0	ENSG00000130119.15	ENSG00000130119	GNL3L	PE243	1	Unique
1	0	ENSG00000130787.13	ENSG00000130787	HIP1R	PE243	1	Unique
1	1	ENSG00000131238.17	ENSG00000131238	PPT1	PE243, MR766	2	Shared
1	1	ENSG00000133624.13	ENSG00000133624	ZNF767P	PE243, MR766	2	Shared
1	0	ENSG00000135723.13	ENSG00000135723	FHOD1	PE243	1	Unique
1	0	ENSG00000136193.16	ENSG00000136193	SCRN1	PE243	1	Unique
1	0	ENSG00000138448.11	ENSG00000138448	ITGAV	PE243	1	Unique
1	0	ENSG00000140948.11	ENSG00000140948	ZCCHC14	PE243	1	Unique
1	0	ENSG00000142173.14	ENSG00000142173	COL6A2	PE243	1	Unique
1	0	ENSG00000143761.15	ENSG00000143761	ARF1	PE243	1	Unique
1	0	ENSG00000144233.9	ENSG00000144233	AMMECR1L	PE243	1	Unique
1	0	ENSG00000145012.13	ENSG00000145012	LPP	PE243	1	Unique

1	1	ENSG00000148180.19	ENSG00000148180	GSN	PE243, MR766	2	Shared
1	1	ENSG00000148248.13	ENSG00000148248	SURF4	PE243, MR766	2	Shared
1	0	ENSG00000149177.12	ENSG00000149177	PTPRJ	PE243	1	Unique
1	0	ENSG00000149639.14	ENSG00000149639	MTCL2	PE243	1	Unique
1	0	ENSG00000158863.21	ENSG00000158863	FHIP2B	PE243	1	Unique
1	0	ENSG00000161021.12	ENSG00000161021	MAML1	PE243	1	Unique
1	0	ENSG00000161618.9	ENSG00000161618	ALDH16A1	PE243	1	Unique
1	0	ENSG00000162924.13	ENSG00000162924	REL	PE243	1	Unique
1	0	ENSG00000164362.18	ENSG00000164362	TERT	PE243	1	Unique
1	0	ENSG00000164609.9	ENSG00000164609	SLU7	PE243	1	Unique
1	0	ENSG00000165704.14	ENSG00000165704	HPRT1	PE243	1	Unique
1	0	ENSG00000170802.15	ENSG00000170802	FOXN2	PE243	1	Unique
1	0	ENSG00000171365.15	ENSG00000171365	CLCN5	PE243	1	Unique
1	0	ENSG00000173542.8	ENSG00000173542	MOB1B	PE243	1	Unique
1	0	ENSG00000173898.11	ENSG00000173898	SPTBN2	PE243	1	Unique
1	0	ENSG00000174669.11	ENSG00000174669	SLC29A2	PE243	1	Unique
1	0	ENSG00000175634.14	ENSG00000175634	RPS6KB2	PE243	1	Unique
1	0	ENSG00000179051.13	ENSG00000179051	RCC2	PE243	1	Unique
1	1	ENSG00000183741.11	ENSG00000183741	CBX6	PE243, MR766	2	Shared
1	0	ENSG00000188064.9	ENSG00000188064	WNT7B	PE243	1	Unique
1	0	ENSG00000196118.11	ENSG00000196118	CFAP119	PE243	1	Unique
1	0	ENSG00000196123.12	ENSG00000196123	MATCAP1	PE243	1	Unique
1	0	ENSG00000197063.10	ENSG00000197063	MAFG	PE243	1	Unique
1	0	ENSG00000197457.9	ENSG00000197457	STMN3	PE243	1	Unique
1	0	ENSG00000197714.8	ENSG00000197714	ZNF460	PE243	1	Unique
1	1	ENSG00000197879.14	ENSG00000197879	MYO1C	PE243, MR766	2	Shared
1	0	ENSG00000204267.13	ENSG00000204267	TAP2	PE243	1	Unique
1	0	ENSG00000206652.1	ENSG00000206652	RNU1-1	PE243	1	Unique
1	1	ENSG00000221420.2	ENSG00000221420	SNORA81	PE243, MR766	2	Shared
1	0	ENSG00000235655.3	ENSG00000235655	H3P6	PE243	1	Unique
1	1	ENSG00000241489.7	ENSG00000241489		PE243, MR766	2	Shared
1	0	ENSG00000248092.7	ENSG00000248092	NNT-AS1	PE243	1	Unique
1	1	ENSG00000263513.5	ENSG00000263513	FAM72C	PE243, MR766	2	Shared
1	0	ENSG00000268942.2	ENSG00000268942	CKS1BP3	PE243	1	Unique
0	1	ENSG00000002834.17	ENSG00000002834	LASP1	MR766	1	Unique
0	1	ENSG00000005022.5	ENSG00000005022	SLC25A5	MR766	1	Unique
0	1	ENSG00000009413.15	ENSG00000009413	REV3L	MR766	1	Unique
0	1	ENSG00000011332.19	ENSG00000011332	DPF1	MR766	1	Unique
0	1	ENSG00000013810.18	ENSG00000013810	TACC3	MR766	1	Unique
0	1	ENSG00000049449.8	ENSG00000049449	RCN1	MR766	1	Unique
0	1	ENSG00000058262.9	ENSG00000058262	SEC61A1	MR766	1	Unique
0	1	ENSG00000069275.12	ENSG00000069275	NUCKS1	MR766	1	Unique
0	1	ENSG00000075089.9	ENSG00000075089	ACTR6	MR766	1	Unique
0	1	ENSG00000075785.12	ENSG00000075785	RAB7A	MR766	1	Unique
0	1	ENSG00000078618.21	ENSG00000078618	NRDC	MR766	1	Unique
0	1	ENSG00000084090.13	ENSG00000084090	STARD7	MR766	1	Unique

0	1	ENSG00000099219.13	ENSG00000099219	ERMP1	MR766	1	Unique
0	1	ENSG00000099246.16	ENSG00000099246	RAB18	MR766	1	Unique
0	1	ENSG00000100393.12	ENSG00000100393	EP300	MR766	1	Unique
0	1	ENSG00000101346.13	ENSG00000101346	POFUT1	MR766	1	Unique
0	1	ENSG00000101464.10	ENSG00000101464	PIGU	MR766	1	Unique
0	1	ENSG00000102763.17	ENSG00000102763	VWA8	MR766	1	Unique
0	1	ENSG00000103202.12	ENSG00000103202	NME4	MR766	1	Unique
0	1	ENSG00000104408.9	ENSG00000104408	EIF3E	MR766	1	Unique
0	1	ENSG00000104687.12	ENSG00000104687	GSR	MR766	1	Unique
0	1	ENSG00000104964.14	ENSG00000104964	TLE5	MR766	1	Unique
0	1	ENSG00000105290.11	ENSG00000105290	APLP1	MR766	1	Unique
0	1	ENSG00000105552.14	ENSG00000105552	BCAT2	MR766	1	Unique
0	1	ENSG00000108187.15	ENSG00000108187	PBLD	MR766	1	Unique
0	1	ENSG00000108423.14	ENSG00000108423	TUBD1	MR766	1	Unique
0	1	ENSG00000110851.11	ENSG00000110851	PRDM4	MR766	1	Unique
0	1	ENSG00000111752.10	ENSG00000111752	PHC1	MR766	1	Unique
0	1	ENSG00000112592.13	ENSG00000112592	TBP	MR766	1	Unique
0	1	ENSG00000112984.11	ENSG00000112984	KIF20A	MR766	1	Unique
0	1	ENSG00000113068.9	ENSG00000113068	PFDN1	MR766	1	Unique
0	1	ENSG00000115183.14	ENSG00000115183	TANC1	MR766	1	Unique
0	1	ENSG00000115993.12	ENSG00000115993	TRAK2	MR766	1	Unique
0	1	ENSG00000116199.11	ENSG00000116199	FAM20B	MR766	1	Unique
0	1	ENSG00000116266.10	ENSG00000116266	STXBP3	MR766	1	Unique
0	1	ENSG00000116489.12	ENSG00000116489	CAPZA1	MR766	1	Unique
0	1	ENSG00000118873.15	ENSG00000118873	RAB3GAP2	MR766	1	Unique
0	1	ENSG00000119471.14	ENSG00000119471	HSDL2	MR766	1	Unique
0	1	ENSG00000119862.12	ENSG00000119862	LGALSL	MR766	1	Unique
0	1	ENSG00000121057.12	ENSG00000121057	AKAP1	MR766	1	Unique
0	1	ENSG00000121406.8	ENSG00000121406	ZNF549	MR766	1	Unique
0	1	ENSG00000124120.10	ENSG00000124120	TTPAL	MR766	1	Unique
0	1	ENSG00000124208.16	ENSG00000124208	PEDS1-UBE2V	MR766	1	Unique
0	1	ENSG00000125319.14	ENSG00000125319	HROB	MR766	1	Unique
0	1	ENSG00000125753.13	ENSG00000125753	VASP	MR766	1	Unique
0	1	ENSG00000126107.14	ENSG00000126107	HECTD3	MR766	1	Unique
0	1	ENSG00000126602.10	ENSG00000126602	TRAP1	MR766	1	Unique
0	1	ENSG00000126822.16	ENSG00000126822	PLEKHG3	MR766	1	Unique
0	1	ENSG00000127481.14	ENSG00000127481	UBR4	MR766	1	Unique
0	1	ENSG00000128654.13	ENSG00000128654	MTX2	MR766	1	Unique
0	1	ENSG00000128908.15	ENSG00000128908	INO80	MR766	1	Unique
0	1	ENSG00000129173.12	ENSG00000129173	E2F8	MR766	1	Unique
0	1	ENSG00000129245.11	ENSG00000129245	FXR2	MR766	1	Unique
0	1	ENSG00000130309.10	ENSG00000130309	COLGALT1	MR766	1	Unique
0	1	ENSG00000131153.8	ENSG00000131153	GINS2	MR766	1	Unique
0	1	ENSG00000131174.4	ENSG00000131174	COX7B	MR766	1	Unique
0	1	ENSG00000131370.15	ENSG00000131370	SH3BP5	MR766	1	Unique
0	1	ENSG00000131653.12	ENSG00000131653	TRAF7	MR766	1	Unique

0	1	ENSG00000131747.14	ENSG00000131747	TOP2A	MR766	1	Unique
0	1	ENSG00000132591.11	ENSG00000132591	ERAL1	MR766	1	Unique
0	1	ENSG00000132680.10	ENSG00000132680	KHDC4	MR766	1	Unique
0	1	ENSG00000132842.13	ENSG00000132842	AP3B1	MR766	1	Unique
0	1	ENSG00000134308.13	ENSG00000134308	YWHAQ	MR766	1	Unique
0	1	ENSG00000134375.10	ENSG00000134375	TIMM17A	MR766	1	Unique
0	1	ENSG00000135245.9	ENSG00000135245	HILPDA	MR766	1	Unique
0	1	ENSG00000135720.12	ENSG00000135720	DYNC1LI2	MR766	1	Unique
0	1	ENSG00000135916.15	ENSG00000135916	ITM2C	MR766	1	Unique
0	1	ENSG00000135976.17	ENSG00000135976	ANKRD36	MR766	1	Unique
0	1	ENSG00000136205.16	ENSG00000136205	TNS3	MR766	1	Unique
0	1	ENSG00000136937.12	ENSG00000136937	NCBP1	MR766	1	Unique
0	1	ENSG00000137076.20	ENSG00000137076	TLN1	MR766	1	Unique
0	1	ENSG00000138107.12	ENSG00000138107	ACTR1A	MR766	1	Unique
0	1	ENSG00000138641.15	ENSG00000138641	HERC3	MR766	1	Unique
0	1	ENSG00000138756.17	ENSG00000138756	BMP2K	MR766	1	Unique
0	1	ENSG00000140395.8	ENSG00000140395	SKIC8	MR766	1	Unique
0	1	ENSG00000141279.15	ENSG00000141279	NPEPPS	MR766	1	Unique
0	1	ENSG00000141959.16	ENSG00000141959	PFKL	MR766	1	Unique
0	1	ENSG00000141971.12	ENSG00000141971	MVB12A	MR766	1	Unique
0	1	ENSG00000143321.18	ENSG00000143321	HDGF	MR766	1	Unique
0	1	ENSG00000143368.9	ENSG00000143368	SF3B4	MR766	1	Unique
0	1	ENSG00000143418.19	ENSG00000143418	CERS2	MR766	1	Unique
0	1	ENSG00000143486.15	ENSG00000143486	EIF2D	MR766	1	Unique
0	1	ENSG00000143862.7	ENSG00000143862	ARL8A	MR766	1	Unique
0	1	ENSG00000144339.11	ENSG00000144339	TMEFF2	MR766	1	Unique
0	1	ENSG00000144736.13	ENSG00000144736	SHQ1	MR766	1	Unique
0	1	ENSG00000146425.10	ENSG00000146425	DYNLT1	MR766	1	Unique
0	1	ENSG00000146574.15	ENSG00000146574	CCZ1B	MR766	1	Unique
0	1	ENSG00000146648.17	ENSG00000146648	EGFR	MR766	1	Unique
0	1	ENSG00000146731.10	ENSG00000146731	CCT6A	MR766	1	Unique
0	1	ENSG00000147133.15	ENSG00000147133	TAF1	MR766	1	Unique
0	1	ENSG00000147905.17	ENSG00000147905	ZCCHC7	MR766	1	Unique
0	1	ENSG00000148175.12	ENSG00000148175	STOM	MR766	1	Unique
0	1	ENSG00000149948.13	ENSG00000149948	HMGA2	MR766	1	Unique
0	1	ENSG00000151239.13	ENSG00000151239	TWF1	MR766	1	Unique
0	1	ENSG00000152270.8	ENSG00000152270	PDE3B	MR766	1	Unique
0	1	ENSG00000153207.14	ENSG00000153207	AHCTF1	MR766	1	Unique
0	1	ENSG00000153815.16	ENSG00000153815	CMIP	MR766	1	Unique
0	1	ENSG00000153944.10	ENSG00000153944	MSI2	MR766	1	Unique
0	1	ENSG00000154945.6	ENSG00000154945	ANKRD40	MR766	1	Unique
0	1	ENSG00000155366.16	ENSG00000155366	RHOC	MR766	1	Unique
0	1	ENSG00000157869.14	ENSG00000157869	RAB28	MR766	1	Unique
0	1	ENSG00000158435.7	ENSG00000158435	CNOT11	MR766	1	Unique
0	1	ENSG00000159128.14	ENSG00000159128	IFNGR2	MR766	1	Unique
0	1	ENSG00000159685.10	ENSG00000159685	CHCHD6	MR766	1	Unique

0	1	ENSG00000160408.14	ENSG00000160408	ST6GALNAC6	MR766	1	Unique
0	1	ENSG00000161999.11	ENSG00000161999	JMJD8	MR766	1	Unique
0	1	ENSG00000163462.17	ENSG00000163462	TRIM46	MR766	1	Unique
0	1	ENSG00000163466.15	ENSG00000163466	ARPC2	MR766	1	Unique
0	1	ENSG00000163527.9	ENSG00000163527	STT3B	MR766	1	Unique
0	1	ENSG00000164430.15	ENSG00000164430	CGAS	MR766	1	Unique
0	1	ENSG00000166881.9	ENSG00000166881	NEMP1	MR766	1	Unique
0	1	ENSG00000166949.15	ENSG00000166949	SMAD3	MR766	1	Unique
0	1	ENSG00000167085.11	ENSG00000167085	PHB1	MR766	1	Unique
0	1	ENSG00000167182.14	ENSG00000167182	SP2	MR766	1	Unique
0	1	ENSG00000168010.10	ENSG00000168010	ATG16L2	MR766	1	Unique
0	1	ENSG00000168028.13	ENSG00000168028	RPSA	MR766	1	Unique
0	1	ENSG00000168710.17	ENSG00000168710	AHCYL1	MR766	1	Unique
0	1	ENSG00000169764.15	ENSG00000169764	UGP2	MR766	1	Unique
0	1	ENSG00000170540.14	ENSG00000170540	ARL6IP1	MR766	1	Unique
0	1	ENSG00000171530.13	ENSG00000171530	TBCA	MR766	1	Unique
0	1	ENSG00000171792.10	ENSG00000171792	RHNO1	MR766	1	Unique
0	1	ENSG00000173757.9	ENSG00000173757	STAT5B	MR766	1	Unique
0	1	ENSG00000175334.7	ENSG00000175334	BANF1	MR766	1	Unique
0	1	ENSG00000176087.14	ENSG00000176087	SLC35A4	MR766	1	Unique
0	1	ENSG00000176890.15	ENSG00000176890	TYMS	MR766	1	Unique
0	1	ENSG00000177169.9	ENSG00000177169	ULK1	MR766	1	Unique
0	1	ENSG00000180228.12	ENSG00000180228	PRKRA	MR766	1	Unique
0	1	ENSG00000180747.15	ENSG00000180747	SMG1P3	MR766	1	Unique
0	1	ENSG00000182310.13	ENSG00000182310	SPACA6	MR766	1	Unique
0	1	ENSG00000184983.9	ENSG00000184983	NDUFA6	MR766	1	Unique
0	1	ENSG00000185252.18	ENSG00000185252	ZNF74	MR766	1	Unique
0	1	ENSG00000187391.19	ENSG00000187391	MAGI2	MR766	1	Unique
0	1	ENSG00000187840.4	ENSG00000187840	EIF4EBP1	MR766	1	Unique
0	1	ENSG00000188428.19	ENSG00000188428	BLOC1S5	MR766	1	Unique
0	1	ENSG00000198042.10	ENSG00000198042	MAK16	MR766	1	Unique
0	1	ENSG00000198677.10	ENSG00000198677	SKIC3	MR766	1	Unique
0	1	ENSG00000198792.12	ENSG00000198792	TMEM184B	MR766	1	Unique
0	1	ENSG00000198804.2	ENSG00000198804	MT-CO1	MR766	1	Unique
0	1	ENSG00000198886.2	ENSG00000198886	MT-ND4	MR766	1	Unique
0	1	ENSG00000198938.2	ENSG00000198938	MT-CO3	MR766	1	Unique
0	1	ENSG00000200312.1	ENSG00000200312	RN7SKP255	MR766	1	Unique
0	1	ENSG00000203668.2	ENSG00000203668	CHML	MR766	1	Unique
0	1	ENSG00000212907.2	ENSG00000212907	MT-ND4L	MR766	1	Unique
0	1	ENSG00000214026.10	ENSG00000214026	MRPL23	MR766	1	Unique
0	1	ENSG00000215559.8	ENSG00000215559	ANKRD20A11	MR766	1	Unique
0	1	ENSG00000227057.9	ENSG00000227057	WDR46	MR766	1	Unique
0	1	ENSG00000228253.1	ENSG00000228253	MT-ATP8	MR766	1	Unique
0	1	ENSG00000232499.2	ENSG00000232499		MR766	1	Unique
0	1	ENSG00000235174.1	ENSG00000235174	RPL39P3	MR766	1	Unique
0	1	ENSG00000237523.1	ENSG00000237523	LINC00857	MR766	1	Unique

0	1	ENSG00000249915.7	ENSG00000249915	PDCD6	MR766	1	Unique
0	1	ENSG00000252010.1	ENSG00000252010	SCARNA5	MR766	1	Unique
0	1	ENSG00000252690.3	ENSG00000252690	NA	MR766	1	Unique
0	1	ENSG00000256269.7	ENSG00000256269	HMBS	MR766	1	Unique
0	1	ENSG00000259075.6	ENSG00000259075	POC1B-GALN'	MR766	1	Unique
0	1	ENSG00000260537.2	ENSG00000260537		MR766	1	Unique
0	1	ENSG00000261553.5	ENSG00000261553		MR766	1	Unique
0	1	ENSG00000262633.2	ENSG00000262633		MR766	1	Unique
0	1	ENSG00000270181.2	ENSG00000270181	BIVM-ERCC5	MR766	1	Unique
0	1	ENSG00000273373.1	ENSG00000273373	NA	MR766	1	Unique
0	1	ENSG00000273590.4	ENSG00000273590		MR766	1	Unique
0	1	ENSG00000273841.4	ENSG00000273841	TAF9	MR766	1	Unique
