# Supplementary figures and images for: Distinct ZIKV strain signatures and type I IFN modulation reveal a protective role of brain endothelial interferon signaling in vitro and in vivo
Source: Front Cell Infect Microbiol. 2025 Dec 3;15:1726007. doi: 10.3389/fcimb.2025.1726007 (PMC12752121; doi:10.3389/fcimb.2025.1726007)

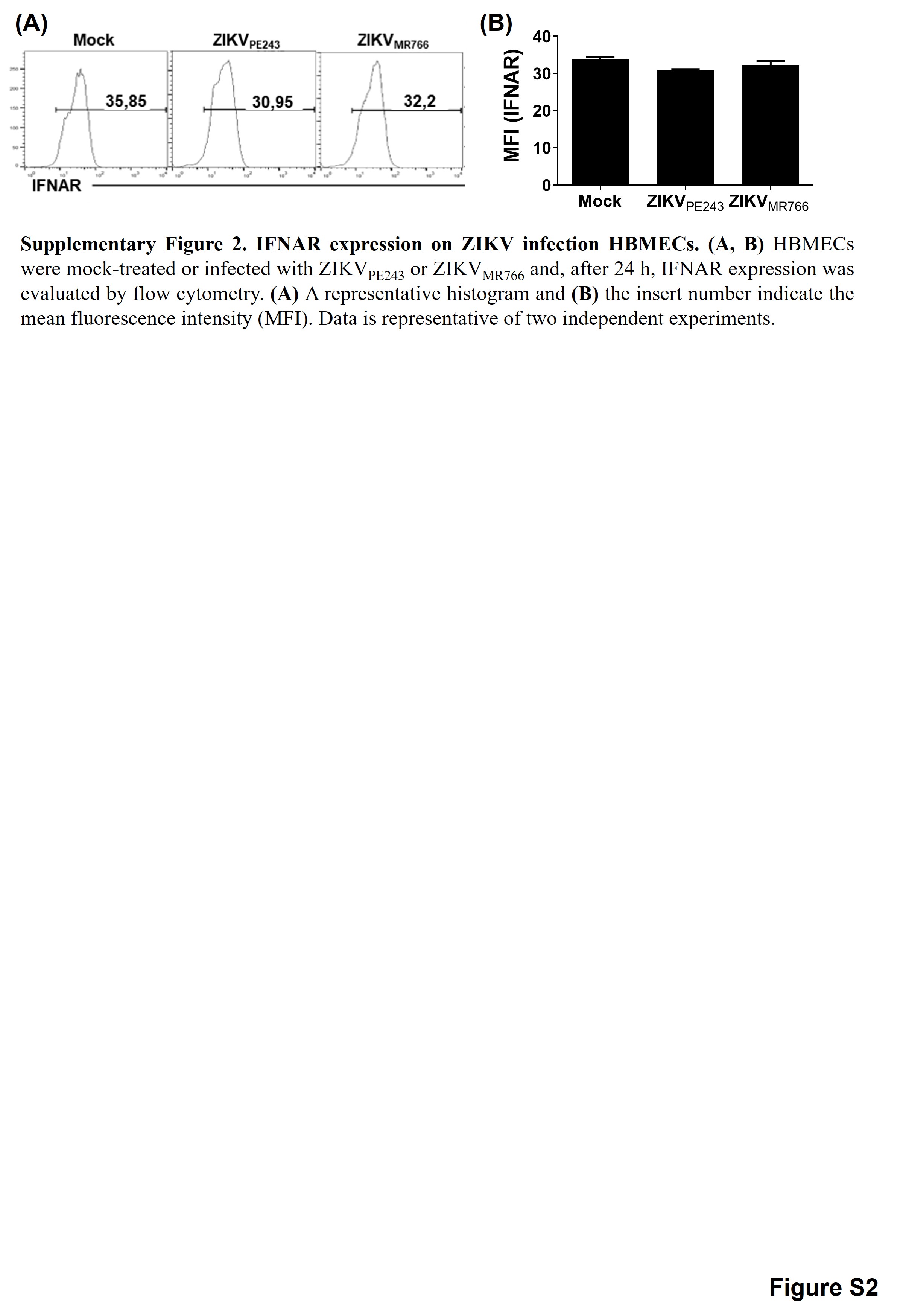

Supplement: Supplementary file 3 [file Image1.jpeg]

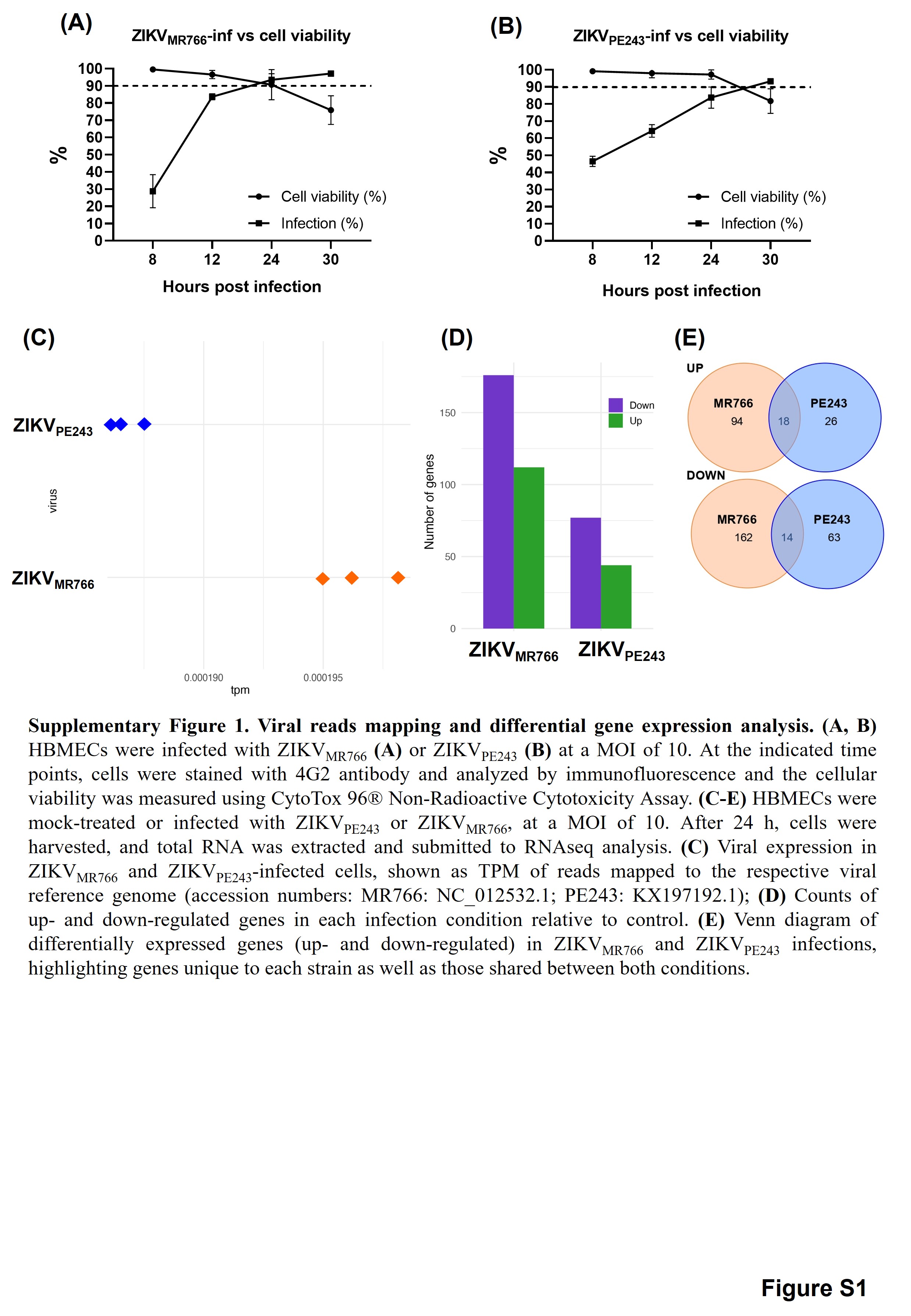

Supplement: Supplementary file 4 [file Image2.jpeg]

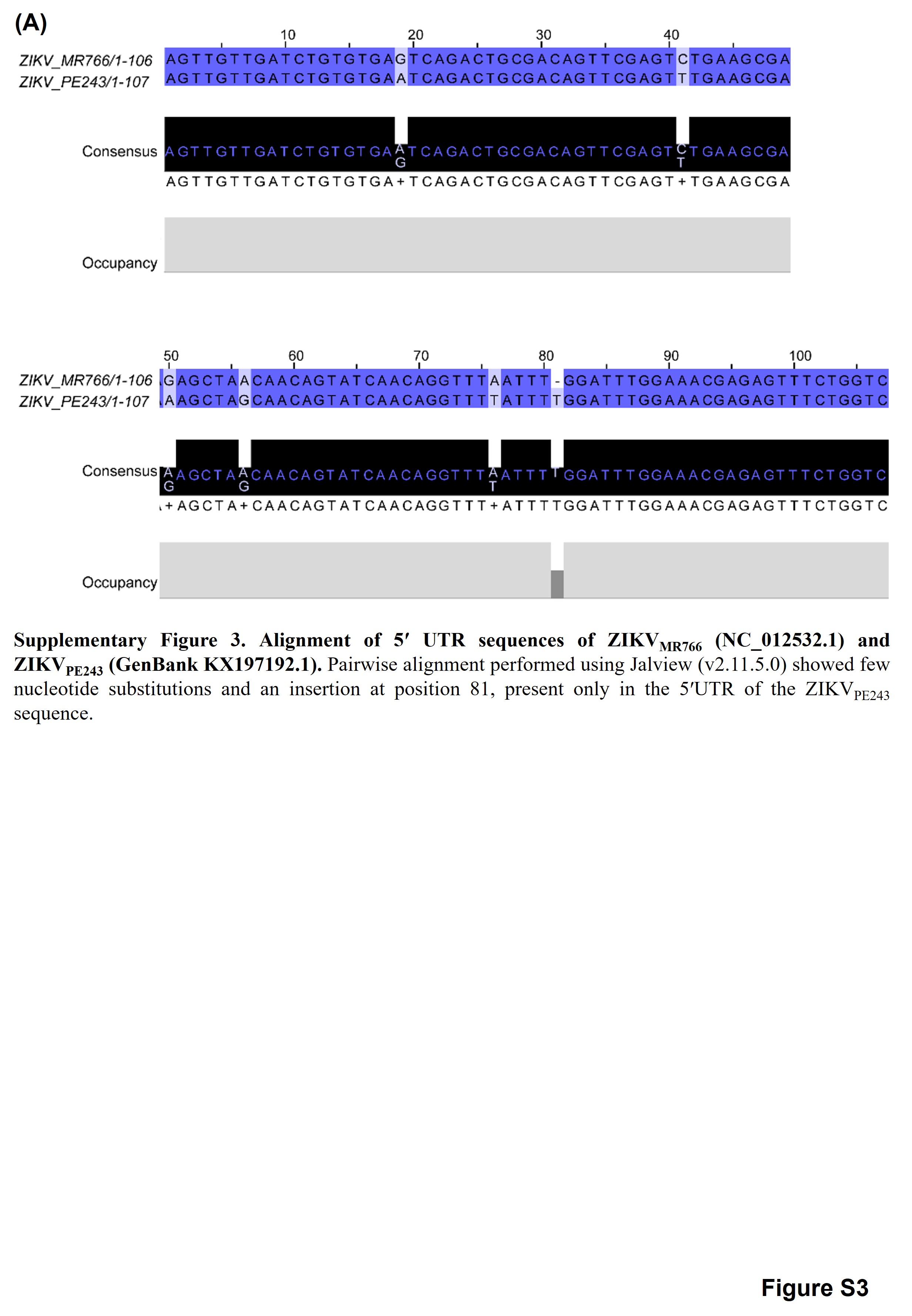

Supplement: Supplementary file 5 [file Image3.jpeg]

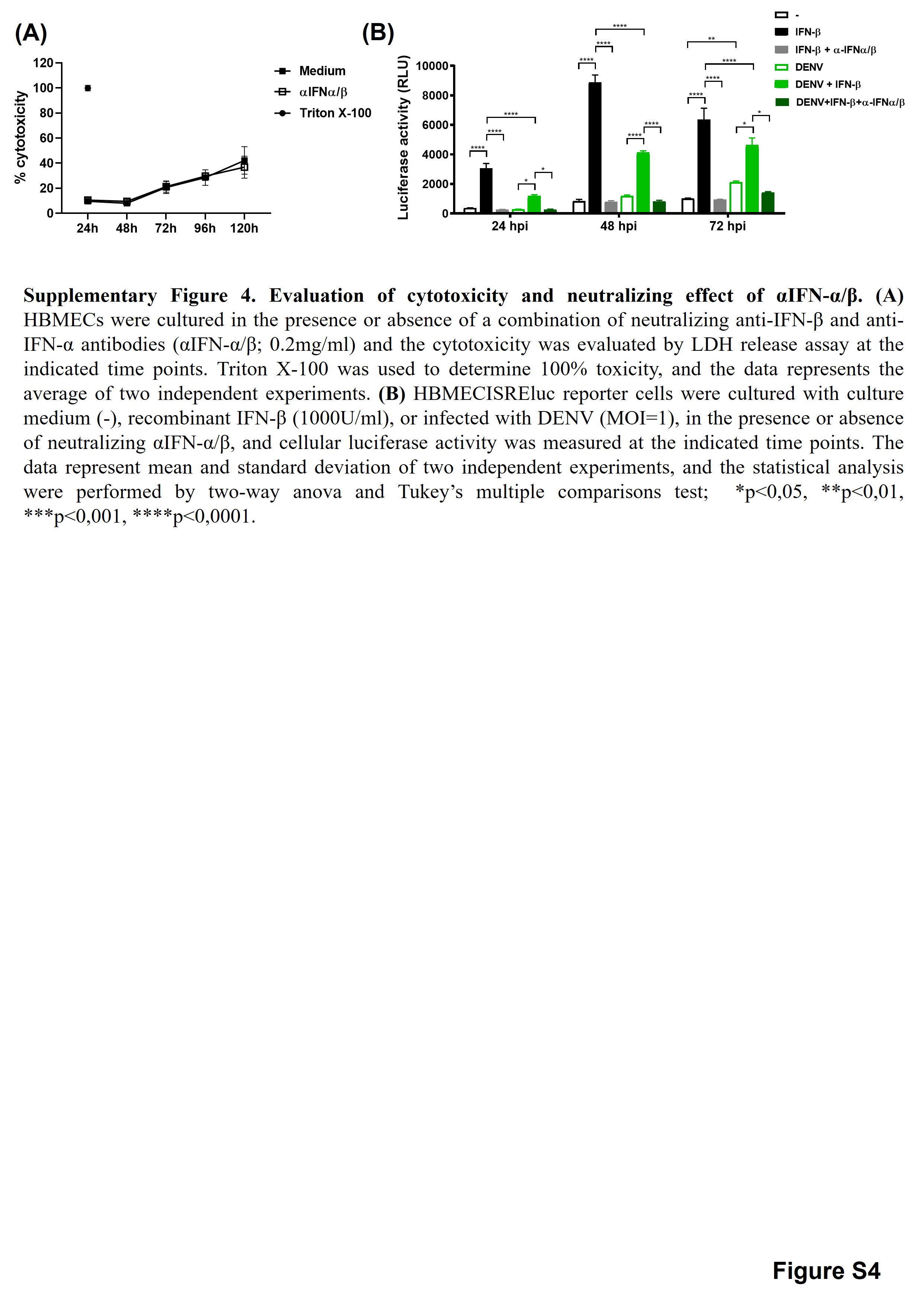

Supplement: Supplementary file 6 [file Image4.jpeg]
